# Life cycle assessment data of French organic agricultural products

**DOI:** 10.1016/j.dib.2021.107356

**Published:** 2021-09-09

**Authors:** Laure Nitschelm, Blanche Flipo, Julie Auberger, Hélène Chambaut, Sylvie Dauguet, Sandrine Espagnol, Armelle Gac, Cécile Le Gall, Caroline Malnoé, Aurélie Perrin, Paul Ponchant, Christel Renaud-Gentié, Aurélie Tailleur, Hayo M.G. van der Werf

**Affiliations:** a UMR SAS, INRAE, Institut Agro, 35000 Rennes, France; b IDELE, 42 rue Georges Morel, 49071 Beaucouze, France; c TERRES INOVIA, Pessac, France; d IFIP, Institut du porc, Le Rheu, France; e IDELE, Monvoisin, Le Rheu, France; f TERRES INOVIA, avenue Lucien Brétignières, Thiverval Grignon, France; g USC 1422 GRAPPE, Ecole Supérieure d'Agricultures (ESA)-INRAE, SFR 4207 QUASAV, 55 rue Rabelais, 49007 Angers, France; h ITAVI, Ploufragan, France; i ARVALIS – Institut du végétal, La Chapelle Saint Sauveur, France

**Keywords:** Organic farming, Environmental assessment, Environmental impacts, Life cycle assessment, France

## Abstract

Environmental data on organic products are needed to assess their environmental performance. The purpose of the ACV Bio project reported here was to generate environmental data as life cycle assessment (LCA) data for a sample of French organic production systems including cropping systems (annual crops, intercrops, forages), grassland, wine grapes, cow milk, calves, beef cattle, sheep, pigs, broilers and eggs. LCA was used to estimate environmental impacts of products from these systems. Recommended uses are to characterize part of the diversity of French organic farming systems and some of their environmental impacts, identify areas for improvement, perform eco-design and sensitivity analysis, and/or make system choices in a given context. However, these data do not represent average French organic products and should not be used as such. The MEANS-InOut web application was used to generate life cycle inventories (LCI). Impact assessment was performed using SimaPro v9 software. The Environmental Footprint 2.0 characterisation method was used to generate LCA data. These data were supplemented with three LCA indicators: cumulative energy demand, land competition (CML-IA non-baseline) and biodiversity loss. Three non-LCA indicators were also calculated for certain systems: diversity of crop families (for cropping systems), agro-ecological infrastructure (for sheep) and pesticide treatment frequency index (for grapes). In total, 173 products were modelled. LCA and non-LCA data are available in the Microsoft® Excel file at Data INRAE (https://doi.org/10.15454/TTR25S). LCI data are available in the AGRIBALYSE database and can be accessed using SimaPro and openLCA software. Farmer-practice data are available on demand.

## Specifications Table

**Table:** 

Subject	Environmental Science
Specific subject area	Life cycle assessment (LCA) data of French organic agricultural products
Type of data	Table
How data were acquired	Data on farmer practices and product yields were collected from farm surveys and existing data bases. Farmer practice data were used as input for simulation models (for details, see section 3) to calculate use of resources and emissions of pollutants to the environment. Data on pollutant emissions and resource use were structured as life cycle inventories using Simapro dedicated life cycle assessment software. The Environmental Footprint characterization method was then used to generate life cycle assessment (LCA) impact indicators in Simapro. LCA indicators were extracted from Simapro to the Microsoft® Excel files associated with this paper.
Data format	Raw
Parameters for data collection	Primary data consisted of farmer practices from real farms, “typical cases” or expert knowledge. Secondary data were collected only from reputable sources: scientific literature or project/expert reports by recognized French institutions (technical institutes, national research institutions and researchers from said institutions, national and regional statistics, national standards).
Description of data collection	The dataset contains LCA data of 173 organic agricultural products. LCA results were expressed per kg of product and per ha of land occupied on-farm and off-farm using the Environmental Footprint 2.0 characterization method. Three other LCA indicators were calculated: cumulative energy demand, land competition (CML-IA non-baseline) and biodiversity loss. For certain systems, three other non-LCA indicators were calculated: diversity of crop families, agro-ecological infrastructure and the pesticide treatment frequency index
Data source location	Institutions: ARVALIS, ESA-Angers, IDELE, IFIP, INRAE, ITAB, ITAVI, TERRES INOVIA City/Town/Region: regional scale (i.e. French administrative region) Country: France
Data accessibility	Repository name: Data INRAE Direct URL to data: https://doi.org/10.15454/TTR25S Instructions for accessing these data: Click on “Access Dataset” then “Download ZIP”.

## Value of the Data

• Data can be used to identify ways to reduce the environmental impacts of organic plant and animal products.
• Any life cycle or agricultural modeller may benefit from these data.
• Data can be used to assess products from French organic farming and quantify several of their environmental impacts per kg of product and per ha of land occupied.
• Data were produced for 173 crop and animal products.
• Data have relevance for France and, more generally, for temperate climate regions. It is recommended, however, to adapt the LCIs to the region concerned.
• Data are available as farmer practices in the MEANS-InOut web application (on demand), as LCI data in SimaPro v9.0 and openLCA software, and as LCA data in the “Data INRAE” repository.

## 1 Data Description

In France, organic farming occupied 2.3 million ha in 2019, which corresponded to 8.5% of agricultural land and 10.4% of farms. Organic farming is often perceived as a way to produce food that has lower environmental impacts than that from conventional farming. Life cycle assessment (LCA) is a methodology that estimates environmental impacts of a product, system or service along all or part of its life cycle. LCA has been used to estimate environmental impacts of organic farming [1], [2], [3], [4], [5], but most of the studies that did so focused on a limited number of products and a few environmental impacts, such as climate change and energy use. Several LCA agricultural databases exist, such as the Agri-footprint and World Food databases, but they contain little or no data on organic farming systems and products. AGRIBALYSE is a French database of life cycle inventories (LCIs) of agricultural products at the farm gate. It originally contained LCIs for 113 products, but only 13 of them came from organic farming. For the most important products from conventional farming, several variants of the same product were described (from different production regions and production systems), thus covering some of the diversity of production systems. For organic products, however, only one variant existed for each product, sometimes to represent a national average. Consequently, the diversity of production systems in organic farming was not apparent, even though it does exist, as in conventional farming. Thus, data on organic farming needed to be generated to assess its environmental impacts. To address this lack of data, the research project ACV Bio, funded by the French agency for ecological transition (ADEME), and the French Ministry for Ecological Transition, was launched. Its main objective was to produce LCI and LCA data on a variety of plant and animal products from French organic farming at the farm gate. The LCI data were integrated in July 2020 into the AGRIBALYSE database and are now available in SimaPro (Pré Consultants, Amersfoort, Netherlands) and openLCA (GreenDelta, Berlin, Germany) software .

Here, we present how we generated LCI and LCA data for a diversity of organic production systems and products, including cropping systems (annual crops, intercrops, forages), grassland, grapes, cow milk, calves, beef cattle, sheep, pigs, broilers and eggs. Data were produced using the Environmental Footprint 2.0 (EF) characterization method [6] for two functional units: 1 kg of product and 1 ha of land occupied. Three additional LCA indicators were calculated: cumulative energy demand (CED) [7], land competition (CML-IA non-baseline) [8] and biodiversity loss [[9], [10], [11]]. For certain systems, three non-LCA indicators were calculated: diversity of crop families (DCF) (for cropping systems) [12], agro-ecological infrastructure (AEI) (for sheep) and the pesticide treatment frequency index (PTFI) (for grapes) [13].

### 1.1 Data generation

LCI data for products of French organic farming were produced: annual crops, intercrops, forages, grassland, grapes, cow milk, calves, beef cattle, sheep, pigs, broilers and eggs (Table 1, see Table S1 for the list of cropping systems). Each annual or perennial crop, forage and intercrop was modelled individually, and some of them were modelled in complete cropping systems (i.e. crop rotations). In total, 173 LCIs were modelled, some of which describe several cases of a given product (from different regions or farms). Hence, a variety of production systems were covered for most products

**Table 1:** Type and number of French organic products considered and total number of life cycle inventories (LCIs).

Type of product	Product	Number of products	Number of LCIs
Plant production			
Annual crops	Barley, blue lupine, chick pea, faba bean, grain maize, oat, rapeseed, soft wheat, sorghum, soya bean, spelt, sunflower, triticale	13	83
Intercrops	Barley/faba bean, barley/pea, triticale/pea, wheat/faba bean, wheat/pea	5	14
Forages and grassland	Grass, alfalfa, silage maize	3	7
Cropping systems	See Table S1	11	11
Grapes	Wine grapes	3	5
Animal production			
Cattle	Cow milk, calf, cull cow from dairy cow system, beef cattle, runner calf, cull cow from beef cow system	6	28
Sheep	Cull ewe, wool, sheep	3	9
Pigs	Cull sow, fattened pig	2	10
Poultry	Egg, cull hen, broiler	3	6

The dataset produced contains 173 LCAs based on these LCIs of organic crop and animal products (Table S2). LCA data for the EF, CED, land competition and biodiversity loss methods, as well as the three non-LCA indicators (DCF, AEI and PTFI), are available in the Microsoft® Excel file available at Data INRAE (https://doi.org/10.15454/TTR25S). The Excel file contains eight tabs (Table 2).

**Table 2:** Description of the eight worksheets in the Microsoft® Excel file. FU = functional unit, n/a = not applicable.

Worksheet name	Content	Characterization method(s)	FU
ReadMe	Description of the data and the other worksheets	n/a	n/a
EF_kg	Environmental impacts	Environmental Footprint 2.0	1 kg
CED_LComp_Biodiv_kg	Environmental impacts	cumulative energy demand, land competition and biodiversity loss	1 kg
EF_ha	Environmental impacts	Environmental Footprint 2.0	1 ha
CED_LComp_Biodiv_ha	Environmental impacts	cumulative energy demand, land competition and biodiversity loss	1 ha
Diversity of crop families	Diversity of crop families for eleven cropping systems	n/a	n/a
Agro-ecological infrastructure	Agro-ecological infrastructure for three sheep systems	n/a	n/a
Pesticide treatment frequency index	Pesticide treatment frequency index for five grape systems	n/a	n/a

## 2 Experimental Design, Materials and Methods

LCA is a methodology that estimates environmental impacts of a product by quantifying the resources consumed and emissions to the environment at several stages of its life cycle. According to the ISO 14040 standard, LCA has four phases: goal and scope definition, inventory analysis, impact assessment and interpretation. In the inventory analysis phase, inputs from the environment (resources used) and outputs to the environment (emissions) associated with the product are listed. In the impact assessment phase, inputs and outputs are transformed into environmental impacts.

### 2.1 Goal and scope definition

The system boundaries defined for each product begin with the extraction of resources and end at the farm gate. The processes included in and excluded from the boundaries are listed in Table 3. We considered two functions to reflect the multi-functionality of agriculture: production and land management. For production, the function was defined as “producing a quantity of an agricultural product in the field, greenhouse or animal production unit, at a specifically defined level of quality or of a defined composition”. For land management, the function was defined as “occupation of agricultural and non-agricultural land for a given amount of time”. The two functional units were thus 1 kg produced (at the reference water content for annual crops, intercrops and silage maize, per kg of dry matter for other forages and per kg of live weight, of milk or of eggs for livestock products) and 1 ha of land occupied during a year. Land occupied includes both “direct” land (on-farm land for crops or animals) and “indirect” land (off-farm land, e.g. for livestock feed, buildings for input production).

**Table 3:** Processes included and excluded in the life cycle inventories.

Products	Processes included	Processes excluded
Cropping systems	All inputs and processes related to crop production: (1) soil cultivation, sowing, weed control, fertilization, pest and pathogen control, harvest; (2) machines and the buildings or areas used to park them; (3) seeds, fertilizers, pesticides, water for irrigation and fuel, as well as their transport to the farm; (4) direct emissions of fuel combustion, tire abrasion and pollutant emissions in the field	Processes that occur after harvest, such as drying, winemaking, sorting and storage, even if they occur on the farm
Annual crops		
Intercrops		
Forages and grassland		
Grapes		
Dairy cows	All inputs and processes related to livestock production: (1) young animals, feed, straw, water (watering and cleaning), fuels and energy, as well as their transport to the farm; (2) buildings and housing as well as infrastructure for milking (for dairy cows); (3) enteric emissions and pollutant emissions due to manure management	Veterinary products and care, artificial insemination, cleaning products and all processes that occur outside the farm (slaughtering, processing, conservation, etc.)
Sheep		
Pigs		
Poultry		
Cattle		

When a process generated several outputs, impacts were allocated among the co-products. Mass allocation was used for intercrop components; 100% allocation to grain and 0% to straw was used for cereals and protein crops; economic allocation was used for co-products of processed crop products such as soya bean and rapeseed; and biophysical allocation was used for animal products (see Koch and Salou [14], p. 82-86, for description of the biophysical allocation).

### 2.2 Life cycle inventories

Farmer-practice data used to create LCIs came from multiple sources. For annual crops and cropping systems, data came from “typical cases”^1^ created by ARVALIS in two research projects: OléAB and ProtéAB. For intercrops, data were based on expert knowledge from TERRES INOVIA, the Chambers of Agriculture of the Hauts de France and Pays de la Loire regions, and INRAE (UMR AGIR). Data for grapes came from real vineyards in the Pays de la Loire and Alsace regions. Each vineyard plot selected for this study represented one of the management types identified in each studied region using a typology. To address the perennial aspect of vineyards, data were collected for 2-4 productive years representative of the climate of the full life cycle, for the 3 first years of the vineyard (including planting), for vineyard destruction and the intercropping period, if relevant. Data for forages and grassland came from typical cases created by ARVALIS (alfalfa, grassland) and from typical cases created by IDELE for sheep and dairy cow systems (grassland). Data for dairy cows came from typical cases created by IDELE and from one real farm. Data for sheep came from a typical case created by IDELE in the Agneaux Bio project. Data for pigs came from real farms. Finally, data for poultry came from technical data compiled by ITAVI.

LCIs were calculated using the MEANS-InOut web application, which is a customised agricultural LCA tool that generates LCIs of agricultural production systems [15]. It contains forms to guide data entry and includes a reference dataset for the main inputs of agri-food systems, analytical models to estimate direct pollutant emissions and resource use, and an export function that generates LCI files ready to be imported in LCA software to calculate impact indicators. Farmer-practice data for each type of production are available on demand in the MEANS-InOut application (contact Julie Auberger, julie.auberger@inrae.fr). Databases used for background processes were AGRIBALYSE 3.0.1 and ecoinvent 3.5. LCIs of animal feeds were created by averaging the crop LCIs and considering processing of crop products when necessary (e.g. to produce soya bean meal). Crop residue dry matter quantities and nitrogen contents were calculated based on the CITEPA method [16], which supplies values that are more accurate for the French situation than the data based on the IPCC 2006 guidelines. For soya bean residue N content the value proposed by CITEPA (2.69%) was very high compared to that of other crop residues. Comparison with measured data confirmed that this value was unrealistic, so instead we used the value proposed by IPCC (0.80%). For alfalfa and grasslands, crop residues were calculated for a 1 year period.

### 2.3 Calculation of emissions

Emissions into the air (ammonia (NH_3_), nitrous oxide (N_2_O), nitrogen oxides (NO_x_), methane (CH_4_), carbon dioxide (CO_2_)), the water (nitrate (NO_3_), phosphorus (P), phosphate (PO_4_), Cd, Cr, Cu, Hg, Ni, Pb, Zn) and the soil (Cd, Cr, Cu, Hg, Ni, Pb, Zn, pesticides) were calculated using models recommended by the AGRIBALYSE methodology [14]. NH_3_ emissions were modelled using a variety of sources. For arable crops, EMEP/EEA 2016 Tier 2 [17] was used to model NH_3_ emissions from organic fertiliser application and livestock excretion on grassland. EMEP/EEA 2009 Tier 2 [18] was used for emission factors of animal excretion in buildings and storage. Finally, models of nitrogen excretion came from CORPEN 2006 [19] and ITAVI 2013 [20] for poultry, RMT 2016 [21] and CORPEN 2003 [22] for pigs, CORPEN 2001 [23] for beef cattle, CORPEN 1999 [24] for dairy cows and AGRIBALYSE methodology [14] for sheep. N_2_O emissions were modelled using IPCC 2019 Tier 1 for crops and grassland, and IPCC 2006 Tier 2 for N_2_O emissions in buildings and storage for livestock production [25,26]. NO_x_ emissions for arable crops and livestock were modelled using EMEP/EEA 2009 Tier 1 [18]. NO_3_ emissions were modelled using Tailleur et al. 2012 [27] for annual crops, the SQCB model [28] for grapes, the DEAC model [29] for grassland and Basset-Mens et al. 2007 [30] for outdoor runs. It was assumed that neither land use change nor changes in farmer practices had occurred in the production systems, consequently soil organic carbon content was assumed to be stable. CH_4_ from animal excretion – in buildings, manure storage, grassland and outdoor runs – and from enteric fermentation in cattle and sheep were modelled using IPCC 2006 Tier 2 [25]. CH_4_ emissions from enteric fermentation were modelled using INRA Feeding System for Ruminants 2018 Tier 3 [14] for ruminants and IPCC 2006 Tier 1 [32] for pigs. CO_2_ emissions from fuel combustion or active substances of pesticides were modelled using ecoinvent® v2 [31]. Finally, P and PO_4_ emissions from leaching, run-off, grazing and grassland were modelled using SALCA-P [38], while Cd, Cr, Cu, Hg, Ni, Pb and Zn emissions to soil and water were modelled using SALCA-ETM [38].

NO_3_ emissions for grassland and alfalfa were modelled using the DEAC model [29]. DEAC considers nitrogen from fertilization and from animal excreta, as well as soil and climate data. For grassland, predicted NO_3_ emissions were higher than those of grassland in the AGRIBALYSE database. Thus, we adjusted these estimates as a function of the ratio of the total amount of nitrogen applied to the NO_3_ emissions from AGRIBALYSE for grassland grazed by conventional cattle (Table S3).

### 2.4 Impact assessment

#### 2.4.1 LCA impact categories

Impact assessment was performed using SimaPro v9 software. We calculated values for 9 of the 16 impacts of the EF method v2.0 [12] (see Table S4 for the detailed list and units of all impact indicators). We only calculated impacts with levels of recommendation I (recommended and satisfactory): Climate change, Ozone depletion, Respiratory inorganics, and II (recommended but in need of some improvements): Ionising radiation (human health), Photochemical ozone formation (human health), Acidification terrestrial and freshwater, Eutrophication terrestrial, Eutrophication freshwater, Eutrophication marine, according to Fazio et al. 2018 [12] . The climate change impact category was adjusted by setting the characterisation factor “CO_2_ in air” to 0, because we assumed that CO_2_ absorbed by plants does not count as carbon sequestration, since it returns to the air within a short period. The “CO_2_ from land transformation” characterisation factor was also set to 0 since we excluded CO_2_ emitted from land transformation.

Other impact categories were used to supplement the dataset. Cumulative Energy Demand v1.11 was used to estimate energy use in MJ throughout the life cycle [13]. Land competition from CML-IA non-baseline v3.04 was used to calculate land occupation in m²year [14]. Finally, we used three biodiversity indicators: Knudsen et al. [15] for temperate annual crops and grassland, Mueller, de Baan and Koellner [16] for tropical annual crops (for soya beans in animal feed), and Koellner and Scholz [17] for grapes. These three indicators are based on species richness of vascular plants and provide characterization factors that differentiate the biodiversity impact of conventional and organic farming (Table 4).

**Table 4:** Characterization factors available in the literature to estimate potential biodiversity loss. Those in bold were used in this study.

Land-use type	Type of production	Knudsen et al. (2017)	Mueller et al. (2014)	Koellner and Scholz (2008)
Temperate annual crops	Organic	**0.21**	0.15	0.36
	Conventional	**0.51**	0.60	0.74
Tropical annual crops	Organic	-	**0.42**	-
	Conventional	-	**0.81**	-
Monocotyledon grasslands	Organic	**-0.16**	-	-
	Conventional	**-0.15**	-	-
Mixed grasslands	Organic	**-0.56**	-	-
	Conventional	**-0.34**	-	-
Grapes	Organic	-	-	**0.42**
	Conventional	-	-	**0.57**

#### 2.4.2 Non-LCA indicators

DCF, calculated for each cropping system, increases as the number of crop families in the cropping system increases and as the distribution of crops among these families becomes more equal (Equation [1]) [18]:

(1) $$\text{CD}=\frac{1}{\sum_{i}{\left(\frac{n_{i}}{N}\right)}^{2}}$$

with n_i_ the number of crops in taxonomic family i and N the number of crops in the cropping system.

The AEI indicator [19], expressed per ha of agricultural land and calculated for sheep production, assesses semi-natural habitats that are managed extensively and not treated with fertilisers or pesticides:

• linear metres of hedges, embankments, low walls, forest edges and copses
• ha of buffer strips, buffer zones, fallow land, permanent grassland, rangeland, heaths, summer pastures and orchards
• m² of ponds and peatlands
• number of isolated trees

Finally, PTFI [19], calculated for grape production, is “the mean number of treatments of commercial pesticide products per hectare, weighted by the ratio of the dose used to the recommended dose”. Table 5 summarizes the LCA and non-LCA indicators calculated for each product.

**Table 5:** Life cycle assessment (LCA) impact characterization methods and non-LCA indicators available for each type of product.

Type of product	LCA impact characterization methods	Non-LCA indicators
Annual crops	EF Method 2.0, Cumulative Energy Demand v1.11, Land competition and Biodiversity loss	None
Cropping systems		Diversity of crop families
Intercrops		None
Forages and grassland		None
Grapes		Pesticide treatment frequency index
Cattle	EF Method 2.0, Cumulative Energy Demand v1.11, Land competition and Biodiversity loss	None
Sheep		Agro-ecological infrastructure
Pigs		None
Poultry		None

## Ethics Statement

The authors declare that creation of these data did not involved the use of human or animal subjects, nor data collection from social media platforms.

## Credit Author Statement

**Laure Nitschelm:** Data curation, Formal analysis, Investigation, Project administration, Investigation, Software, Supervision, Validation, Writing – original draft. **Blanche Flipo:** Data curation, Formal analysis, Writing – review & editing. **Julie Auberger:** Software, Writing – review & editing. **Hélène Chambaut:** Conceptualization, Formal analysis, Methodology, Resources, Validation, Writing – review & editing. **Sylvie Dauguet:** Conceptualization, Formal analysis, Methodology, Resources, Validation, Writing – review & editing. **Sandrine Espagnol:** Conceptualization, Formal analysis, Methodology, Resources, Validation, Writing – review & editing. **Armelle Gac:** Conceptualization, Formal analysis, Methodology, Resources, Validation, Writing – review & editing. **Cécile Le Gall:** Formal analysis, Resources, Validation, Writing – review & editing. **Caroline Malnoé:** Software. **Aurélie Perrin:** Conceptualization, Formal analysis, Methodology, Resources, Validation, Writing – review & editing. **Paul Ponchant:** Conceptualization, Formal analysis, Methodology, Resources, Validation, Writing – review & editing. **Christel Renaud-Gentié:** Conceptualization, Formal analysis, Methodology, Resources, Validation, Writing – review & editing. **Aurélie Tailleur:** Conceptualization, Formal analysis, Methodology, Resources, Validation, Writing – review & editing. **Hayo M.G. van der Werf:** Conceptualization, Formal analysis, Funding acquisition, Methodology, Project administration, Supervision, Validation, Writing – original draft, Writing – review & editing.

## Declaration of Competing Interest

The authors declare that they have no known competing financial interests or personal relationships which have or could be perceived to have influenced the work reported in this article.

## Appendix Supplementary materials

Image, application 1

## Footnotes

1 A typical case describes, using a set of technical and economic or environmental indicators, normal operation of a farm, for a given system and conditions. A typical case illustrates the operation and performance of a typical production system.
