# Supplementary material for: Life cycle assessment data of French organic agricultural products
Source: Data Brief. 2021 Sep 9;38:107356. doi: 10.1016/j.dib.2021.107356 (PMC8446808; doi:10.1016/j.dib.2021.107356)
Supplement: Supplementary file 1 [file mmc1.docx]

**Article Title**

Life cycle assessment data of French organic agricultural products – Supplementary information

**Authors**

Laure Nitschelm^1^, Blanche Flipo^1^, Julie Auberger^1^, Hélène Chambaut^2^, Sylvie Dauguet^3^, Sandrine Espagnol^4^, Armelle Gac^5^, Cécile Le Gall^6^, Caroline Malnoé^1^, Aurélie Perrin^7^, Paul Ponchant^8^, Christel Renaud-Gentié^7^, Aurélie Tailleur^9^, Hayo M.G. van der Werf^1^

**Affiliations**

^1^UMR SAS, INRAE, Institut Agro, 35000 Rennes, France

^2^IDELE, 42 rue Georges Morel, 49071 Beaucouze, France

^3^TERRES INOVIA, Pessac, France

^4^IFIP, Institut du porc, Le Rheu, France

^5^IDELE, Monvoisin, Le Rheu, France

^6^TERRES INOVIA, avenue Lucien Brétignières, Thiverval Grignon, France

^7^USC 1422 GRAPPE, Ecole Supérieure d’Agricultures (ESA)-INRAE, SFR 4207 QUASAV, 55 rue Rabelais, 49007 Angers, France

^8^ITAVI, Ploufragan, France

^9^ARVALIS – Institut du végétal, La Chapelle Saint Sauveur, France

**Corresponding author(s)**

Hayo M.G. van der Werf (hayo.van-der-werf@inrae.fr).

**Tables**

Table S1. Description of cropping systems, including duration in years. “/”: intercrop; “()”: catch crop.

| **Name** | **Duration** | **Cropping system description** |
| --- | --- | --- |
| Cropping system no. 1, organic, at farm gate/FR U | 7 | Alfalfa/Orchardgrass (3 years) - Winter wheat - Winter wheat - Winter triticale/Pea - (Sorghum) - Spring barley |
| Cropping system no. 2, organic, at farm gate/FR U | 9 | Ryegrass/Clover (3 years) - Winter wheat - Winter oat - (Phacelia) Spring faba bean - Winter wheat - (Mustard) Spring barley - Winter triticale/Pea |
| Cropping system no. 3, organic, at farm gate/FR U | 9 | Alfalfa (3 years) - Winter wheat - Winter triticale - (White clover) Spring barley - Winter faba bean - Winter wheat - Spring barley |
| Cropping system no. 4, organic, at farm gate/FR U | 3 | Winter wheat - (Clover) Soybean - (Vetch-Oat) Soybean |
| Cropping system no. 5, organic, at farm gate/FR U | 6 | Winter wheat - Soybean - Soybean - Winter triticale - Sunflower - Chickpea |
| Cropping system no. 6, organic, at farm gate/FR U | 3 | Winter wheat - (Vetch-Oat) Maize - Winter faba bean |
| Cropping system no. 7, organic, at farm gate/FR U | 5 | Winter wheat - Winter triticale/Pea - (Mustard) Spring faba bean - Winter wheat - (Vetch-Rye) Sunflower |
| Cropping system no. 8, organic, at farm gate/FR U | 9 | Alfalfa (3 years) - Winter wheat - (Vetch-Oat) Maize - Winter faba bean - Winter triticale - (Vetch-Oat) Sunflower - Winter barley |
| Cropping system no. 9, organic, at farm gate/FR U | 9 | Alfalfa (3 years) - Winter wheat - Winter barley - Winter faba bean - Winter wheat - (Vetch-Oat) Sunflower - Winter wheat |
| Cropping system no. 10, organic, at farm gate/FR U | 3 | Winter wheat - (Vetch-Oat) Maize - Soybean |
| Cropping system no. 11, organic, at farm gate/FR U | 6 | Alfalfa (3 years) - Winter wheat - Winter wheat - (Vetch-Oat) Sunflower |

Table S2. Names and descriptions of the life cycle inventories (LCIs).

| **LCI name** | **LCI description** |
| --- | --- |
| Grain maize, organic, system no. 1, at farm gate/FR U | Comment: These data represent a typical case and are not representative of a national or regional average  system no. 1: Crop from cropping system no. 6; year 2 in crop rotation; grain yield 8.2 t/ha, standard moisture content of 28%; located in Pays de la Loire, France; production potential of the soil is good; irrigated; cropping system is fertilized 2 years out of 3 with compost from poultry manure and dried laying-hen droppings; data are from a typical case from the CASDAR project ProtéAB. |
| Grain maize, organic, system no. 2, at farm gate/FR U | Comment: These data represent a typical case and are not representative of a national or regional average  system no. 2: Crop from cropping system no. 8; year 5 in crop rotation; grain yield 8.8 t/ha, standard moisture content of 28%; located in Poitou-Charentes, France; production potential of the soil is average/good; cropping system is irrigated; cropping system is fertilized 3 years out of 9 with compost from poultry manure; data are from a typical case from the CASDAR project ProtéAB. |
| Grain maize, organic, system no. 3, at farm gate/FR U | Comment: These data represent a typical case and are not representative of a national or regional average  system no. 3: Crop from cropping system no. 10; year 2 in crop rotation; grain yield 10.0 t/ha; located in Rhône-Alpes, standard moisture content of 28%, France; production potential of the soil is good; cropping system is irrigated; cropping system is fertilized 2 years out of 3 with compost from poultry manure and meat meal; data are from a typical case from the CASDAR project OléAB. |
| Spelt, organic, at farm gate/FR U | Comment: this LCI was created from expert knowledge: Personal communication of A. Tailleur (Arvalis) for yield, mechanisation and operations.  Technical data from Chambre d'agriculture Lorraine (2013) and Aube. |
| Spring barley, from intercrop, organic, system no. 1, at farm gate/FR U | Comment: These data represent a typical case and are not representative of a national or regional average  system no. 1: Crop from spring barley and spring faba bean intercrop system no. 1; intercrop was modeled without considering the cropping system; sorted grain yield 1.5 t/ha, standard moisture content of 14%; intercrop is composed of 63% protein crop-37% cereal; intercrop aim: to produce protein crop; intercrop is non-irrigated; intercrop is non-fertilized; intercrop system built by a group of experts. |
| Spring barley, from intercrop, organic, system no. 2, at farm gate/FR U | Comment: These data represent a typical case and are not representative of a national or regional average  system no. 2: Crop from spring pea and spring barley intercrop system no. 1; Intercrop was modeled without considering the cropping system; sorted grain yield 2.0 t/ha, standard moisture content of 14%; intercrop is composed of 50% protein crop-50% cereal; intercrop aim: to produce protein crop; intercrop is non-irrigated; intercrop is non-fertilized; intercrop system built by a group of experts. |
| Spring barley, organic, system no. 1, at farm gate/FR U | Comment: These data represent a typical case and are not representative of a national or regional average  system no. 1: Crop from cropping system no. 2; year 8 in crop rotation; grain yield 3.0 t/ha, standard moisture content of 15%; located in Brittany, France; production potential of the soil is average/good; cropping system is non-irrigated; cropping system is fertilized 3 years out of 9 with bovine manure and compost from poultry manure; data are from a typical case from the CASDAR project ProtéAB. |
| Spring barley, organic, system no. 2, at farm gate/FR U | Comment: These data represent a typical case and are not representative of a national or regional average  system no. 2: Crop from cropping system no. 1; year 7 in crop rotation; grain yield 1.8 t/ha, standard moisture content of 15%; located in Bourgogne, France; production potential of the soil is low/average; cropping system is non-irrigated; cropping system is fertilized 3 years out of 7 with bovine manure; data are from a typical case from the CASDAR project ProtéAB. |
| Spring barley, organic, system no. 3, at farm gate/FR U | Comment: These data represent a typical case and are not representative of a national or regional average  system no. 3: Crop from cropping system no. 3; year 6 in crop rotation; grain yield 3.6 t/ha, standard moisture content of 15%; located in Ile-de-France, France; production potential of the soil is good; cropping system is non-irrigated; cropping system is fertilized 4 years out of 10 with compost from poultry manure and concentrated residues of beetroot distillation; data are from a typical case from the CASDAR project RotAB. |
| Spring barley, organic, system no. 4, at farm gate/FR U | Comment: These data represent a typical case and are not representative of a national or regional average  system no. 4: Crop from cropping system no. 3; year 9 in crop rotation; grain yield 3.6 t/ha, standard moisture content of 15%; located in Ile-de-France, France; production potential of the soil is good; cropping system is non-irrigated; cropping system is fertilized 4 years out of 10 with compost from poultry manure and concentrated residues of beetroot distillation; data are from a typical case from the CASDAR project RotAB. |
| Spring wheat, from intercrop, organic, system no. 1, at farm gate/FR U | Comment: These data represent a typical case and are not representative of a national or regional average  system no. 1: Crop from spring faba bean and spring wheat intercrop system no. 1; intercrop was modeled without considering the cropping system; sorted grain yield 1.5 t/ha, standard moisture content of 15%; intercrop is composed of 63% protein crop-37% cereal; intercrop aim: to produce protein crop; intercrop is non-irrigated; intercrop is non-fertilized; intercrop system built by a group of experts. |
| Spring wheat, from intercrop, organic, system no. 2, at farm gate/FR U | Comment: These data represent a typical case and are not representative of a national or regional average  system no. 2: Crop from spring pea and spring wheat intercrop system no. 1; intercrop was modeled without considering the cropping system; sorted grain yield 2.0 t/ha, standard moisture content of 15%; intercrop is composed of 50% protein crop-50% cereal; intercrop aim: to produce protein crop; intercrop is non-irrigated; intercrop is non-fertilized; intercrop system built by a group of experts. |
| Triticale grain, from intercrop, organic, system no. 1, at farm gate/FR U | Comment: These data represent a typical case and are not representative of a national or regional average  system no. 1: Crop from spring pea and triticale intercrop system no. 1; intercrop was modeled without considering the cropping system; sorted grain yield 3.5 t/ha, standard moisture content of 15%; intercrop is composed of 22% protein crop-78% cereal; intercrop aim: to produce animal feed; intercrop is non-irrigated; intercrop is fertilized; intercrop system built by a group of experts. |
| Triticale grain, from intercrop, organic, system no. 2, at farm gate/FR U | Comment: These data represent a typical case and are not representative of a national or regional average  system no. 2: Crop from triticale grain and winter pea intercrop system no. 2; intercrop from cropping system no. 2; year 9 in crop rotation; sorted grain yield 2.7 t/ha, standard moisture content of 15%; intercrop is composed of 67.5% protein crop-32.5% cereal; located in Brittany, France; production potential of the soil is average/good; intercrop is non-irrigated; intercrop is non-fertilized; cropping system is fertilized 3 years out of 9 with bovine manure and compost from poultry manure; data are from a typical case from the CASDAR project ProtéAB. |
| Triticale grain, from intercrop, organic, system no. 3, at farm gate/FR U | Comment: These data represent a typical case and are not representative of a national or regional average  system no. 3: Crop from triticale grain and winter pea intercrop system no. 3; intercrop from cropping system no. 1; year 6 in crop rotation; grain yield 1.6 t/ha, standard moisture content of 15%; intercrop is composed of 36% protein crop-64% cereal; located in Bourgogne, France; production potential of the soil is low/average; intercrop is non-irrigated; intercrop is non-fertilized; cropping system is non-irrigated; cropping system is fertilized 3 years out of 7 with bovine manure; data are from a typical case from the CASDAR project ProtéAB. |
| Triticale grain, from intercrop, organic, system no. 4, at farm gate/FR U | Comment: These data represent a typical case and are not representative of a national or regional average  system no. 4: Crop from Triticale grain and winter pea intercrop system no. 4; intercrop from cropping system no. 7; year 2 in crop rotation; grain yield 2.03 t/ha, standard moisture content of 15%; intercrop is composed of 77% protein crop-23% cereal; located in Pays de la Loire, France; production potential of the soil is average; intercrop is non-irrigated; intercrop is non-fertilized; cropping system is non-irrigated; cropping system is fertilized 3 years out of 5 with compost from poultry manure; data are from a typical case from the CASDAR project ProtéAB. |
| Triticale grain, organic, system no. 1, at farm gate/FR U | Comment: These data represent a typical case and are not representative of a national or regional average  system no. 1: Crop from cropping system no. 3; year 5 in crop rotation; grain yield 4.1 t/ha, standard moisture content of 15%; located in Ile-de-France, France; production potential of the soil is good; cropping system is non-irrigated; cropping system is fertilized 4 years out of 10 with compost from poultry manure and concentrated residues of beetroot distillation; data are from a typical case from the CASDAR project RotAB. |
| Triticale grain, organic, system no. 2, at farm gate/FR U | Comment: These data represent a typical case and are not representative of a national or regional average  system no. 2: Crop from cropping system no. 5; year 4 in crop rotation; grain yield 2.8 t/ha, standard moisture content of 15%; located in Midi-Pyrénées, France; production potential of the soil is average; cropping system is non-irrigated; cropping system is fertilized 3 years out of 6 with 9-5-0 fertilizer usable in organic agriculture; data are from a typical case from Agribenchmark. |
| Triticale grain, organic, system no. 3, at farm gate/FR U | Comment: These data represent a typical case and are not representative of a national or regional average  system no. 3: Crop from cropping system no. 8; year 7 in crop rotation; grain yield 4.0 t/ha, standard moisture content of 15%; located in Poitou-Charentes, France; production potential of the soil is average/good; cropping system is irrigated; cropping system is fertilized 3 years out of 9 with compost from poultry manure; data are from a typical case from the CASDAR project ProtéAB. |
| Winter barley, from intercrop, organic, system no. 1, at farm gate/FR U | Comment: These data represent a typical case and are not representative of a national or regional average  system no. 1: Crop from winter faba bean and winter barley intercrop system no. 1; intercrop was modeled without considering the cropping system; unsorted grain yield 1.5 t/ha, standard moisture content of 15%; 63% protein crop-37% cereal; intercrop aim: to produce protein crop; intercrop is non-irrigated; intercrop is non-fertilized; intercrop system built by a group of experts. |
| Winter barley, from intercrop, organic, system no. 2, at farm gate/FR U | Comment: These data represent a typical case and are not representative of a national or regional average  system no. 2: Crop from winter barley and winter pea intercrop system no. 1; intercrop was modeled without considering the cropping system; unsorted grain yield 2.0 t/ha, standard moisture content of 15%; 50% protein crop-50% cereal; intercrop aim: to produce protein crop; intercrop is non-irrigated; intercrop is non-fertilized; intercrop system built by a group of experts. |
| Winter barley, organic, system no. 1, at farm gate/FR U | Comment: These data represent a typical case and are not representative of a national or regional average  system no. 1: Crop from cropping system no. 8; year 9 in crop rotation; grain yield 3.5 t/ha, standard moisture content of 15%; located in Poitou-Charentes, France; production potential of the soil is average/good; cropping system is irrigated; cropping system is fertilized 3 years out of 9 with compost from poultry manure; data are from a typical case from the CASDAR project ProtéAB. |
| Winter barley, organic, system no. 2, at farm gate/FR U | Comment: These data represent a typical case and are not representative of a national or regional average  system no. 2: Crop from cropping system no. 9; year 5 in crop rotation; grain yield 3.5 t/ha, standard moisture content of 15%; located in Poitou-Charentes, France; production potential of the soil is average/good; cropping system is non-irrigated; cropping system is fertilized 2 years out of 9 with compost from poultry manure; data are from a typical case from the CASDAR project ProtéAB. |
| Winter oat, organic, system no. 1, at farm gate/FR U | Comment: These data represent a typical case and are not representative of a national or regional average  system no. 1: Crop from cropping system no. 2; year 5 in crop rotation; grain yield 3.0 t/ha, standard moisture content of 12%; located in Brittany, France; production potential of the soil is average/good; cropping system is non-irrigated; cropping system is fertilized 3 years out of 9 with bovine manure and compost from poultry manure; data are from a typical case from the CASDAR project ProtéAB. |
| Winter wheat, from intercrop, organic, system no. 1, at farm gate/FR U | Comment: These data represent a typical case and are not representative of a national or regional average  system no. 1: Crop from winter wheat and winter faba bean intercrop system no. 1; intercrop was modeled without considering the cropping system; unsorted grain yield 1.0 t/ha, standard moisture content of 15%; 75% protein crop-25% cereal; intercrop aim: to produce high-protein wheat; intercrop is non-irrigated; intercrop is fertilized; intercrop system built by a group of experts. |
| Winter wheat, from intercrop, organic, system no. 2, at farm gate/FR U | Comment: These data represent a typical case and are not representative of a national or regional average  system no. 2: Crop from winter wheat and winter faba bean intercrop system no. 2; intercrop was modeled without considering the cropping system; unsorted grain yield 1.5 t/ha, standard moisture content of 15%; 63% protein crop-37% cereal; intercrop aim: to produce protein crop; intercrop is non-irrigated; intercrop is non-fertilized; intercrop system built by a group of experts. |
| Winter wheat, from intercrop, organic, system no. 3, at farm gate/FR U | Comment: These data represent a typical case and are not representative of a national or regional average  system no. 3: Crop from winter wheat and winter pea intercrop system no. 1; intercrop was modeled without considering the cropping system; unsorted grain yield 1.0 t/ha, standard moisture content of 15%; 75% protein crop-25% cereal; intercrop aim: to produce high-protein wheat; intercrop is non-irrigated; intercrop is fertilized; intercrop system built by a group of experts. |
| Winter wheat, from intercrop, organic, system no. 4, at farm gate/FR U | Comment: These data represent a typical case and are not representative of a national or regional average  system no. 4: Crop from winter wheat and winter pea intercrop system no. 2; intercrop was modeled without considering the cropping system; unsorted grain yield 2.0 t/ha, standard moisture content of 15%; 50% protein crop-50% cereal; intercrop aim: to produce protein crop; intercrop is non-irrigated; intercrop is non-fertilized; intercrop system built by a group of experts. |
| Winter wheat, organic, system no. 1, at farm gate/FR U | Comment: These data represent a typical case and are not representative of a national or regional average  system no. 1: Crop from cropping system no. 2; year 4 in crop rotation; grain yield 4.0 t/ha, standard moisture content of 15%; located in Brittany, France; production potential of the soil is average/good; cropping system is non-irrigated; cropping system is fertilized 3 years out of 9 with bovine manure and compost from poultry manure; data are from a typical case from the CASDAR project ProtéAB. |
| Winter wheat, organic, system no. 2, at farm gate/FR U | Comment: These data represent a typical case and are not representative of a national or regional average  system no. 2: Crop from cropping system no. 2; year 7 in crop rotation; grain yield 3.5 t/ha, standard moisture content of 15%; located in Brittany, France; production potential of the soil is average/good; cropping system is non-irrigated; cropping system is fertilized 3 years out of 9 with bovine manure and compost from poultry manure; data are from a typical case from the CASDAR project ProtéAB. |
| Winter wheat, organic, system no. 3, at farm gate/FR U | Comment: These data represent a typical case and are not representative of a national or regional average  system no. 3: Crop from cropping system no. 1; year 4 in crop rotation; grain yield 2.5 t/ha, standard moisture content of 15%; located in Bourgogne, France; production potential of the soil is low/average; cropping system is non-irrigated; cropping system is fertilized 3 years out of 7 with bovine manure; data are from a typical case from the CASDAR project ProtéAB. |
| Winter wheat, organic, system no. 4, at farm gate/FR U | Comment: These data represent a typical case and are not representative of a national or regional average  system no. 4: Crop from cropping system no. 1; year 5 in crop rotation; grain yield 2.0 t/ha, standard moisture content of 15%; located in Bourgogne, France; production potential of the soil is low/average; cropping system is non-irrigated; cropping system is fertilized 3 years out of 7 with bovine manure; data are from a typical case from the CASDAR project ProtéAB. |
| Winter wheat, organic, system no. 5, at farm gate/FR U | Comment: These data represent a typical case and are not representative of a national or regional average  system no. 5: Crop from cropping system no. 3; year 4 in crop rotation; grain yield 4.6 t/ha, standard moisture content of 15%; located in Ile-de-France, France; production potential of the soil is good; cropping system is non-irrigated; cropping system is fertilized 4 years out of 10 with compost from poultry manure and concentrated residues of beetroot distillation; data are from a typical case from the CASDAR project RotAB. |
| Winter wheat, organic, system no. 6, at farm gate/FR U | Comment: These data represent a typical case and are not representative of a national or regional average  system no. 6: Crop from cropping system no. 3; year 8 in crop rotation; grain yield 4.0 t/ha, standard moisture content of 15%; located in Ile-de-France, France; production potential of the soil is good; cropping system is non-irrigated; cropping system is fertilized 4 years out of 10 with compost from poultry manure and concentrated residues of beetroot distillation; data are from a typical case from the CASDAR project RotAB. |
| Winter wheat, organic, system no. 7, at farm gate/FR U | Comment: These data represent a typical case and are not representative of a national or regional average  system no. 7: Crop from cropping system no. 4; year 1 in crop rotation; grain yield 3.3 t/ha, standard moisture content of 15%; located in Midi-Pyrénées, France; production potential of the soil is average; cropping system is irrigated; cropping system is fertilized 1 years out of 3 with compost from poultry manure and 10-0-0 fertilizer usable in organic agriculture; data are from a typical case from Agribenchmark. |
| Winter wheat, organic, system no. 8, at farm gate/FR U | Comment: These data represent a typical case and are not representative of a national or regional average  system no. 8: Crop from cropping system no. 5; year 1 in crop rotation; grain yield 2.6 t/ha, standard moisture content of 15%; located in Midi-Pyrénées, France; production potential of the soil is average; cropping system is non-irrigated; cropping system is fertilized 3 years out of 6 with 9-5-0 fertilizer usable in organic agriculture; data are from a typical case from Agribenchmark. |
| Winter wheat, organic, system no. 9, at farm gate/FR U | Comment: These data represent a typical case and are not representative of a national or regional average  system no. 9: Crop from cropping system no. 6; year 1 in crop rotation; grain yield 3.8 t/ha, standard moisture content of 15%; located in Pays de la Loire, France; production potential of the soil is good; cropping system is irrigated; cropping system is fertilized 2 years out of 3 with compost from poultry manure and dried laying-hen droppings; data are from a typical case from the CASDAR project ProtéAB. |
| Winter wheat, organic, system no. 10, at farm gate/FR U | Comment: These data represent a typical case and are not representative of a national or regional average  system no. 10: Crop from cropping system no. 7; year 1 in crop rotation; grain yield 2.9 t/ha, standard moisture content of 15%; located in Pays de la Loire, France; production potential of the soil is average; cropping system is non-irrigated; cropping system is fertilized 3 years out of 5 with compost from poultry manure; data are from a typical case from the CASDAR project ProtéAB. |
| Winter wheat, organic, system no. 11, at farm gate/FR U | Comment: These data represent a typical case and are not representative of a national or regional average  system no. 11: Crop from cropping system no. 7; year 4 in crop rotation; grain yield 3.2 t/ha, standard moisture content of 15%; located in Pays de la Loire, France; production potential of the soil is average; cropping system is non-irrigated; cropping system is fertilized 3 years out of 5 with compost from poultry manure; data are from a typical case from the CASDAR project ProtéAB. |
| Winter wheat, organic, system no. 12, at farm gate/FR U | Comment: These data represent a typical case and are not representative of a national or regional average  system no. 12: Crop from cropping system no. 8; year 4 in crop rotation; grain yield 4.5 t/ha, standard moisture content of 15%; located in Poitou-Charentes, France; production potential of the soil is average/good; cropping system is irrigated; cropping system is fertilized 3 years out of 9 with compost from poultry manure; data are from a typical case from the CASDAR project ProtéAB. |
| Winter wheat, organic, system no. 13, at farm gate/FR U | Comment: These data represent a typical case and are not representative of a national or regional average  system no. 13: Crop from cropping system no. 9; year 4 in crop rotation; grain yield 2.8 t/ha, standard moisture content of 15%; located in Poitou-Charentes, France; production potential of the soil is average/good; cropping system is non-irrigated; cropping system is fertilized 2 years out of 9 with compost from poultry manure; data are from a typical case from the CASDAR project ProtéAB. |
| Winter wheat, organic, system no. 14, at farm gate/FR U | Comment: These data represent a typical case and are not representative of a national or regional average  system no. 14: Crop from cropping system no. 9; year 7 in crop rotation; grain yield 2.8 t/ha, standard moisture content of 15%; located in Poitou-Charentes, France; production potential of the soil is average/good; cropping system is non-irrigated; cropping system is fertilized 2 years out of 9 with compost from poultry manure; data are from a typical case from the CASDAR project ProtéAB. |
| Winter wheat, organic, system no. 15, at farm gate/FR U | Comment: These data represent a typical case and are not representative of a national or regional average  system no. 15: Crop from cropping system no. 9; year 9 in crop rotation; grain yield 3.5 t/ha, standard moisture content of 15%; located in Poitou-Charentes, France; production potential of the soil is average/good; cropping system is non-irrigated; cropping system is fertilized 2 years out of 9 with compost from poultry manure; data are from a typical case from the CASDAR project ProtéAB. |
| Winter wheat, organic, system no. 16, at farm gate/FR U | Comment: These data represent a typical case and are not representative of a national or regional average  system no. 16: Crop from cropping system no. 10; year 1 in crop rotation; grain yield 4.5 t/ha, standard moisture content of 15%; located in Rhône-Alpes, France; production potential of the soil is good; cropping system is irrigated; cropping system is fertilized 2 years out of 3 with compost from poultry manure and meat meal; data are from a typical case from the CASDAR project OléAB. |
| Winter wheat, organic, system no. 17, at farm gate/FR U | Comment: These data represent a typical case and are not representative of a national or regional average  system no. 17: Crop from cropping system no. 11; year 4 in crop rotation; grain yield 3.5 t/ha, standard moisture content of 15%; located in Rhône-Alpes, France; production potential of the soil is good; cropping system is non-irrigated; cropping system is fertilized 2 years out of 6 with potassium fertilizer usable in organic agriculture and compost from poultry manure; data are from a typical case from the CASDAR project OléAB. |
| Winter wheat, organic, system no. 18, at farm gate/FR U | Comment: These data represent a typical case and are not representative of a national or regional average  system no. 18: Crop from cropping system no. 11; year 5 in crop rotation; grain yield 3.5 t/ha, standard moisture content of 15%; located in Rhône-Alpes, France; production potential of the soil is good; cropping system is non-irrigated; cropping system is fertilized 2 years out of 6 with potassium fertilizer usable in organic agriculture and compost from poultry manure; data are from a typical case from the CASDAR project OléAB. |
| Cropping system no. 1, organic, at farm gate/FR U | Comment: These data represent a typical case and are not representative of a national or regional average.  system no. 1: Crop rotation is Alfalfa and dactyl intercrop (3 years)/Winter wheat/Winter wheat/Triticale and pea intercrop/Sorghum (catch crop that is harvested)/Spring barley; rotation duration is 7 years; average annual yield is 5,53 t of dry matter/ha; located in Bourgogne, France; production potential of the soil is low/average; non-irrigated; fertilized 3 years out of 7 with bovine manure; data are from a typical case from the CASDAR project ProtéAB. |
| Cropping system no. 2, organic, at farm gate/FR U | Comment: These data represent a typical case and are not representative of a national or regional average.  system no. 2: Crop rotation is Rye grass and red clover intercrop (3 years)/Winter wheat/Winter oat/(catch crop) Spring faba bean/Winter wheat/Spring barley/Winter triticale and pea intercrop; rotation duration is 9 years; average annual yield is 3,22 t of dry matter/ha; located in Brittany, France; production potential of the soil is average/good; non-irrigated; fertilized 3 years out of 9 with bovine manure and compost from poultry manure; data are from a typical case from the CASDAR project ProtéAB. |
| Cropping system no. 3, organic, at farm gate/FR U | Comment: These data represent a typical case and are not representative of a national or regional average.  system no. 3: Crop rotation is Alfalfa (3 years)/Winter wheat/Winter triticale/(catch crop) Spring barley/Winter faba bean/Winter wheat/(catch crop) Spring barley; rotation duration is 9 years; average annual yield is 5,27 t of dry matter/ha; located in Ile-de-France, France; production potential of the soil is good; non-irrigated; fertilized 4 years out of 10 with compost from poultry manure and concentrated residues of beetroot distillation; data are from a typical case from the CASDAR project RotAB. |
| Cropping system no. 4, organic, at farm gate/FR U | Comment: These data represent a typical case and are not representative of a national or regional average.  system no. 4: Crop rotation is Winter wheat/Soybean/(catch crop) Soybean; rotation duration is 3 years; average annual yield is 2,54 t of dry matter/ha; located in Midi-Pyrénées, France; production potential of the soil is average; irrigated; fertilized 1 years out of 3 with compost from poultry manure and 10-0-0 fertilizer usable in organic agriculture; data are from a typical case from Agribenchmark. |
| Cropping system no. 5, organic, at farm gate/FR U | Comment: These data represent a typical case and are not representative of a national or regional average.  system no. 6: Crop rotation is Winter Wheat/Soybean/Soybean/Winter Triticale/Sunflower/Chickpea; rotation duration is 6 years; average annual yield is 1,72 t of dry matter/ha; located in Midi-Pyrénées, France; production potential of the soil is average; non-irrigated; fertilized 3 years out of 6 with 9-5-0 fertilizer usable in organic agriculture; data are from a typical case from Agribenchmark. |
| Cropping system no. 6, organic, at farm gate/FR U | Comment: These data represent a typical case and are not representative of a national or regional average.  system no. 6: Crop rotation is Winter wheat/(catch crop) Maize/Winter faba bean; rotation duration is 3 years; average annual yield is 3,83 t of dry matter/ha; located in Pays de la Loire, France; production potential of the soil is good; irrigated; fertilized 2 years out of 3 with compost from poultry manure and dried laying-hen droppings; data are from a typical case from the CASDAR project ProtéAB. |
| Cropping system no. 7, organic, at farm gate/FR U | Comment: These data represent a typical case and are not representative of a national or regional average.  system no. 7: Crop rotation is Winter wheat/Triticale and pea intercrop/(catch crop) Spring faba bean/Winter wheat/(catch crop) Sunflower; rotation duration is 5 years; average annual yield is 2,33 t of dry matter/ha; located in Pays de la Loire, France; production potential of the soil is average; non-irrigated; fertilized 3 years out of 5 with compost from poultry manure; data are from a typical case from the CASDAR project ProtéAB. |
| Cropping system no. 8, organic, at farm gate/FR U | Comment: These data represent a typical case and are not representative of a national or regional average.  system no. 8: Crop rotation is Alfalfa (3 years)/Winter wheat/ (catch crop) Maize/Winter faba bean/Winter triticale/(catch crop) Sunflower/Winter barley; rotation duration is 9 years; average annual yield is 4.45 t of dry matter/ha; located in Poitou-Charentes, France; production potential of the soil is average/good; irrigated; fertilized 3 years out of 9 with compost from poultry manure; data are from a typical case from the CASDAR project ProtéAB. |
| Cropping system no. 9, organic, at farm gate/FR U | Comment: These data represent a typical case and are not representative of a national or regional average.  system no. 9: Crop rotation is Alfalfa (3 years)/Winter wheat/Winter barley/Winter faba bean/Winter wheat/(catch crop) Sunflower/Winter wheat; rotation duration is 9 years; average annual yield is 4.87 t of dry matter/ha; located in Poitou-Charentes, France; production potential of the soil is average/good; non-irrigated; fertilized 2 years out of 9 with compost from poultry manure; data are from a typical case from the CASDAR project ProtéAB. |
| Cropping system no. 10, organic, at farm gate/FR U | Comment: These data represent a typical case and are not representative of a national or regional average.  system no. 10: Crop rotation is Winter wheat/(catch crop) Maize/Soybean; rotation duration is 3 years; average annual yield is 4.82 t of dry matter/ha; located in Rhône-Alpes, France; production potential of the soil is good; irrigated; fertilized 2 years out of 3 with compost from poultry manure and meat meal; data are from a typical case from the CASDAR project OléAB. |
| Cropping system no. 11, organic, at farm gate/FR U | Comment: These data represent a typical case and are not representative of a national or regional average.  system no. 11: Crop rotation is Alfalfa (3 years)/Winter wheat/Winter wheat/(catch crop) Sunflower; rotation duration is 6 years; average annual yield is 4,96 t of dry matter/ha; located in Rhône-Alpes, France; production potential of the soil is good; non-irrigated; fertilized 2 years out of 6 with potassium fertilizer usable in organic agriculture and compost from poultry manure; data are from a typical case from the CASDAR project OléAB. |
| Grape, organic, PDO, dry white wine, system no. 1, at vineyard gate/FR U | Comment: These data represent a real farm and are not representative of a national or regional average  system no. 1: Protected Designation of Origin (PDO); dry white wine; located in Alsace; yield: 11.25 t/ha.year; organic production; grape variety: Riesling; mean treatment frequency index per year: 6; mean copper application: 3.39 kg/ha.year; 3 years of non-productive phase; vine lifetime: 43 years; vintages chosen to represent the whole vine lifetime: 2013, 2015 and 2017. |
| Grape, organic, PDO, dry white wine, system no. 2, at vineyard gate/FR U | Comment: These data represent a real farm and are not representative of a national or regional average  system no. 2: Protected Designation of Origin (PDO); dry white wine; located in Alsace; yield: 7.35 t/ha.year; biodynamic production; grape variety: Riesling; mean treatment frequency index per year: 7; mean copper application: 0.76 kg/ha.year; 3 years of non-productive phase; vine lifetime: 43 years; vintages chosen to represent the whole vine lifetime: 2013, 2015 and 2017. |
| Grape, organic, PDO, dry white wine, system no. 3, at vineyard gate/FR U | Comment: These data represent a real farm and are not representative of a national or regional average  system no. 3: Protected Designation of Origin (PDO); dry white wine; located in Low Loire Valley; yield: 6.57 t/ha.year; organic production; grape variety: Melon de Bourgogne; mean treatment frequency index per year: 9; mean copper application: 2.36 kg/ha.year; 3 years of non-productive phase; vine lifetime: 33 years; vintages chosen to represent the whole vine lifetime: 2013 and 2017. |
| Grape, organic, PDO, dry white wine, system no. 4, at vineyard gate/FR U | Comment: These data represent a real farm and are not representative of a national or regional average  system no. 4: Protected Designation of Origin (PDO); dry white wine; located in Middle Loire Valley; yield: 7.10 t/ha.year; organic production; grape variety: Chenin Blanc; mean treatment frequency index per year: 5; mean copper application: 1.75 kg/ha.year; 3 years of non-productive phase; vine lifetime: 33 years; vintages chosen to represent the whole vine lifetime: 2010, 2011, 2012 and 2013. |
| Grape, organic, PDO, dry white wine, system no. 5, at vineyard gate/FR U | Comment: These data represent a real farm and are not representative of a national or regional average  system no. 5: Protected Designation of Origin (PDO); dry white wine; located in Middle Loire Valley; yield: 2.25 t/ha.year; organic production; grape variety: Chenin Blanc; mean treatment frequency index per year: 9; mean copper application: 1.08 kg/ha.year; 3 years of non-productive phase; vine lifetime: 33 years; vintages chosen to represent the whole vine lifetime: 2010, 2011, 2012 and 2013. |
| Spring faba bean and spring barley intercrop, organic, system no. 1, at farm gate/FR U | Comment: These data represent a typical case and are not representative of a national or regional average  system no. 1: Intercrop was modeled without considering the cropping system; unsorted grain yield 4.0 t/ha, standard moisture content of 14% for protein crop and 15% for cereal; 63% protein crop-37% cereal; intercrop aim: to produce protein crop; intercrop is non-irrigated; intercrop is non-fertilized; intercrop system built by a group of experts. |
| Spring faba bean and spring wheat intercrop, organic, system no. 1, at farm gate/FR U | Comment: These data represent a typical case and are not representative of a national or regional average  system no. 1: Intercrop was modeled without considering the cropping system; unsorted grain yield 4.0 t/ha, standard moisture content of 14% for protein crop and 15% for cereal; 63% protein crop-37% cereal; intercrop aim: to produce protein crop; intercrop is non-irrigated; intercrop is non-fertilized; intercrop system built by a group of experts. |
| Spring pea and spring barley intercrop, organic, system no. 1, at farm gate/FR U | Comment: These data represent a typical case and are not representative of a national or regional average  system no. 1: Intercrop was modeled without considering the cropping system; unsorted grain yield 4.0 t/ha, standard moisture content of 14% for protein crop and 15% for cereal; 50% protein crop-50% cereal; intercrop aim: to produce protein crop; intercrop is non-irrigated; intercrop is non-fertilized; intercrop system built by a group of experts. |
| Spring pea and spring wheat intercrop, organic, system no. 1, at farm gate/FR U | Comment: These data represent a typical case and are not representative of a national or regional average  system no. 1: Intercrop was modeled without considering the cropping system; unsorted grain yield 4.0 t/ha, standard moisture content of 14% for protein crop and 15% for cereal; 50% protein crop-50% cereal; intercrop aim: to produce protein crop; intercrop is non-irrigated; intercrop is non-fertilized; intercrop system built by a group of experts. |
| Winter faba bean and winter barley intercrop, organic, system no. 1, at farm gate/FR U | Comment: These data represent a typical case and are not representative of a national or regional average  system no. 1: Intercrop was modeled without considering the cropping system; unsorted grain yield 4.0 t/ha, standard moisture content of 14% for protein crop and 15% for cereal; 63% protein crop-37% cereal; intercrop aim: to produce protein crop; intercrop is non-irrigated; intercrop is non-fertilized; intercrop system built by a group of experts. |
| Winter faba bean and winter wheat intercrop, organic, system no. 1, at farm gate/FR U | Comment: These data represent a typical case and are not representative of a national or regional average  system no. 1: Intercrop was modeled without considering the cropping system; unsorted grain yield 4.0 t/ha, standard moisture content of 14% for protein crop and 15% for cereal; 75% protein crop-25% cereal; intercrop aim: to produce high-protein wheat; intercrop is non-irrigated; intercrop is fertilized; intercrop system built by a group of experts. |
| Winter faba bean and winter wheat intercrop, organic, system no. 2, at farm gate/FR U | Comment: These data represent a typical case and are not representative of a national or regional average  system no. 1: Intercrop was modeled without considering the cropping system; unsorted grain yield 4.0 t/ha, standard moisture content of 14% for protein crop and 15% for cereal; 63% protein crop-37% cereal; intercrop aim: to produce protein crop; intercrop is non-irrigated; intercrop is non-fertilized; intercrop system built by a group of experts. |
| Winter pea and triticale grain intercrop, organic, system no. 1, at farm gate/FR U | Comment: These data represent a typical case and are not representative of a national or regional average  system no. 1: Intercrop was modeled without considering the cropping system; unsorted grain yield 4.5 t/ha, standard moisture content of 14% for protein crop and 15% for cereal; 22% protein crop-78% cereal; intercrop aim: to produce animal feed; intercrop is non-irrigated; intercrop is fertilized; intercrop system built by a group of experts. |
| Winter pea and triticale grain intercrop, organic, system no. 2, at farm gate/FR U | Comment: These data represent a typical case and are not representative of a national or regional average  system no. 2: Intercrop from cropping system no. 2; year 9 in crop rotation; unsorted grain yield 4.0 t/ha, standard moisture content of 14% for protein crop and 15% for cereal; intercrop is composed of 36% protein crop-64% cereal; located in Brittany, France; production potential of the soil is average/good; intercrop is non-irrigated; cropping system is non-irrigated; intercrop is non-fertilized; cropping system is fertilized 3 years out of 9 with bovine manure and compost from poultry manure; data are from a typical case from the CASDAR project ProtéAB. |
| Winter pea and triticale grain intercrop, organic, system no. 3, at farm gate/FR U | Comment: These data represent a typical case and are not representative of a national or regional average  system no. 3: Intercrop from cropping system no. 1; year 6 in crop rotation; grain yield 2.5 t/ha, standard moisture content of 14% for protein crop and 15% for cereal; intercrop is composed of 36% protein crop-64% cereal; located in Bourgogne, France; production potential of the soil is low/average; intercrop is non-irrigated; intercrop is non-fertilized; cropping system is non-irrigated; cropping system is fertilized 3 years out of 7 with bovine manure; data are from a typical case from the CASDAR project ProtéAB. |
| Winter pea and triticale grain intercrop, organic, system no. 4, at farm gate/FR U | Comment: These data represent a typical case and are not representative of a national or regional average  system no. 4: Intercrop from cropping system no. 7; year 2 in crop rotation; grain yield 10.73 t/ha, standard moisture content of 14% for protein crop and 15% for cereal; intercrop is composed of 77% protein crop-23% cereal; located in Pays de la Loire, France; production potential of the soil is average; intercrop is non-irrigated; intercrop is non-fertilized; cropping system is non-irrigated; cropping system is fertilized 3 years out of 5 with compost from poultry manure; data are from a typical case from the CASDAR project ProtéAB. |
| Winter pea and winter barley intercrop, organic, system no. 1, at farm gate/FR U | Comment: These data represent a typical case and are not representative of a national or regional average  system no. 2: Intercrop was modeled without considering the cropping system; unsorted grain yield 4.0 t/ha, standard moisture content of 14% for protein crop and 15% for cereal; 50% protein crop-50% cereal; intercrop aim: to produce protein crop; intercrop is non-irrigated; intercrop is non-fertilized; intercrop system built by a group of experts. |
| Winter pea and winter wheat intercrop, organic, system no. 1, at farm gate/FR U | Comment: These data represent a typical case and are not representative of a national or regional average  system no. 1: Intercrop was modeled without considering the cropping system; unsorted grain yield 4.0 t/ha, standard moisture content of 14% for protein crop and 15% for cereal; 75% protein crop-25% cereal; intercrop aim: to produce high-protein wheat; intercrop is non-irrigated; intercrop is fertilized; intercrop system built by a group of experts. |
| Winter pea and winter wheat intercrop, organic, system no. 2, at farm gate/FR U | Comment: These data represent a typical case and are not representative of a national or regional average  system no. 2: Intercrop was modeled without considering the cropping system; unsorted grain yield 4.0 t/ha, standard moisture content of 14% for protein crop and 15% for cereal; 50% protein crop-50% cereal; intercrop aim: to produce protein crop; intercrop is non-irrigated; intercrop is non-fertilized; intercrop system built by a group of experts. |
| Chickpea, organic, system no. 1, at farm gate/FR U | Comment: These data represent a typical case and are not representative of a national or regional average  system no. 1: Crop from cropping system no. 5; year 6 in crop rotation; grain yield 1.5 t/ha, standard moisture content of 15%; located in Midi-Pyrénées, France; production potential of the soil is average; cropping system is non-irrigated; cropping system is fertilized 3 years out of 6 with 9-5-0 fertilizer usable in organic agriculture; data are from a typical case from Agribenchmark. |
| Soybean grain, organic, at farm gate/CN U | Comment: These data comes from litterature. Yield: 2.788 t/ha, standard moisture content of 14%  Sampling procedure: Yield, fertilization, seed quantity, diesel consumption and % of crop residue were adapted from "Knudsen MT, Yu-Hui Q, Luo Yan, Halberg N, 2010. Environmental assessment of organic soybean (Glycine max.) imported from China to Denmark: a case study. J Cleaner Production 18: 1431-1439".  Sowing and harvesting date were supposed to be, respectively, at the end of April and at the begining of september. Mecanisation was only considered for sowing and harvesting. |
| Soybean grain, organic, at farm gate/IN U | Comment: These data comes from litterature. Yield: 1.09 t/ha, standard moisture content of 14%  Sampling procedure: Yield, sowing and harvesting date and crop rotation were adapted from "Aulakh CS, Ravisankar N, 2017. Organic farming in Indian context: a perspective. Agric. Res. J. 54 (2): 149-164" and "Ramesh K, AK Patra et AK Biswas, 2017. Best Management Practices for Soybean under Soybean-Wheat System to Minimize the Impact of Climate Change. Indian Journal of Fertilisers, Vol. 13 (2): 42-55."  Fertilization, seed quantity and diesel consumption were adapted from "Knudsen MT, Yu-Hui Q, Luo Yan, Halberg N, 2010. Environmental assessment of organic soybean (Glycine max.) imported from China to Denmark: a case study. J Cleaner Production 18: 1431-1439". |
| Soybean grain, organic, system no. 1, at farm gate/FR U | Comment: These data represent a typical case and are not representative of a national or regional average  system no. 1: Crop from cropping system no. 4; year 2 in crop rotation; grain yield 2.8 t/ha, standard moisture content of 14%; located in Midi-Pyrénées, France; production potential of the soil is average; cropping system is irrigated; cropping system is fertilized 1 years out of 3 with compost from poultry manure and 10-0-0 fertilizer usable in organic agriculture; data are from a typical case from Agribenchmark. |
| Soybean grain, organic, system no. 2, at farm gate/FR U | Comment: These data represent a typical case and are not representative of a national or regional average  system no. 2: Crop from cropping system no. 4; year 3 in crop rotation; grain yield 2.8 t/ha, standard moisture content of 14%; located in Midi-Pyrénées, France; production potential of the soil is average; cropping system is irrigated; cropping system is fertilized 1 years out of 3 with compost from poultry manure and 10-0-0 fertilizer usable in organic agriculture; data are from a typical case from Agribenchmark. |
| Soybean grain, organic, system no. 3, at farm gate/FR U | Comment: These data represent a typical case and are not representative of a national or regional average  system no. 3: Crop from cropping system no. 5; year 2 in crop rotation; grain yield 2.8 t/ha, standard moisture content of 14%; located in Midi-Pyrénées, France; production potential of the soil is average; cropping system is non-irrigated; cropping system is cropping system is fertilized 3 years out of 6 with 9-5-0 fertilizer usable in organic agriculture; data are from a typical case from Agribenchmark. |
| Soybean grain, organic, system no. 4, at farm gate/FR U | Comment: These data represent a typical case and are not representative of a national or regional average  system no. 4: Crop from cropping system no. 5; year 3 in crop rotation; grain yield 2.8 t/ha, standard moisture content of 14%; located in Midi-Pyrénées, France; production potential of the soil is average; cropping system is non-irrigated; cropping system is fertilized 3 years out of 6 with 9-5-0 fertilizer usable in organic agriculture; data are from a typical case from Agribenchmark. |
| Soybean grain, organic, system no. 5, at farm gate/FR U | Comment: These data represent a typical case and are not representative of a national or regional average  system no. 5: Crop from cropping system no. 10; year 3 in crop rotation; grain yield 4.0 t/ha, standard moisture content of 14%; located in Rhône-Alpes, France; production potential of the soil is good; cropping system is irrigated; cropping system is fertilized 2 years out of 3 with compost from poultry manure and meat meal; data are from a typical case from the CASDAR project OléAB. |
| Blue lupine, organic, at farm gate/FR U | Comment: this LCI was created from expert knowledge.  Sampling procedure: Personal communication from AgroBioPinault (Journée Proléobio 29 mars 2019, ITAB et Terre Inovia). Technical data from Chambre d'Agriculture de Bretagne (2004) - Agrobio Bretagne (2010) |
| Spring faba bean, from intercrop, organic, system no. 1, at farm gate/FR U | Comment: These data represent a typical case and are not representative of a national or regional average  system no. 1: Crop from spring barley and spring faba bean intercrop system no. 1; intercrop was modeled without considering the cropping system; sorted grain yield 2.5 t/ha, standard moisture content of 14%; intercrop is composed of 63% protein crop-37% cereal; intercrop aim: to produce protein crop; intercrop is non-irrigated; intercrop is non-fertilized; intercrop system built by a group of experts. |
| Spring faba bean, from intercrop, organic, system no. 2, at farm gate/FR U | Comment: These data represent a typical case and are not representative of a national or regional average  system no. 2: Crop from spring faba bean and spring wheat intercrop system no. 1; intercrop was modeled without considering the cropping system; sorted grain yield 2.5 t/ha, standard moisture content of 14%; intercrop is composed of 63% protein crop-37% cereal; intercrop aim: to produce protein crop; intercrop is non-irrigated; intercrop is non-fertilized; intercrop system built by a group of experts. |
| Spring faba bean, organic, system no. 1, at farm gate/FR U | Comment: These data represent a typical case and are not representative of a national or regional average  system no. 1: Crop from cropping system no. 2; year 6 in crop rotation; grain yield 3.0 t/ha, standard moisture content of 15%; located in Brittany, France; production potential of the soil is average/good; cropping system is non-irrigated; cropping system is fertilized 3 years out of 9 with bovine manure and compost from poultry manure; data are from a typical case from the CASDAR project ProtéAB. |
| Spring faba bean, organic, system no. 2, at farm gate/FR U | Comment: These data represent a typical case and are not representative of a national or regional average  system no. 2: Crop from cropping system no. 7; year 3 in crop rotation; grain yield 2.3 t/ha, standard moisture content of 15%; located in Pays de la Loire, France; production potential of the soil is average; cropping system is non-irrigated; cropping system is fertilized 3 years out of 5 with compost from poultry manure; data are from a typical case from the CASDAR project ProtéAB. |
| Spring pea, from intercrop, organic, system no. 1, at farm gate/FR U | Comment: These data represent a typical case and are not representative of a national or regional average  system no. 1: Crop from spring pea and spring barley intercrop system no. 1; intercrop was modeled without considering the cropping system; sorted grain yield 2.0 t/ha, standard moisture content of 14%; intercrop is composed of 50% protein crop-50% cereal; intercrop aim: to produce protein crop; intercrop is non-irrigated; intercrop is non-fertilized; intercrop system built by a group of experts. |
| Spring pea, from intercrop, organic, system no. 2, at farm gate/FR U | Comment: These data represent a typical case and are not representative of a national or regional average  system no. 2: Crop from spring pea and spring wheat intercrop system no. 1; intercrop was modeled without considering the cropping system; sorted grain yield 2.0 t/ha, standard moisture content of 14%; intercrop is composed of 50% protein crop-50% cereal; intercrop aim: to produce protein crop; intercrop is non-irrigated; intercrop is non-fertilized; intercrop system built by a group of experts. |
| Winter faba bean, from intercrop, organic, system no. 1, at farm gate/FR U | Comment: These data represent a typical case and are not representative of a national or regional average  system no. 1: Crop from winter faba bean and winter barley intercrop system no. 1; intercrop was modeled without considering the cropping system; unsorted grain yield 2.5 t/ha, standard moisture content of 14%; 63% protein crop-37% cereal; intercrop aim: to produce protein crop; intercrop is non-irrigated; intercrop is non-fertilized; intercrop system built by a group of experts. |
| Winter faba bean, from intercrop, organic, system no. 2, at farm gate/FR U | Comment: These data represent a typical case and are not representative of a national or regional average  system no. 2: Crop from winter wheat and winter faba bean intercrop system no. 1; intercrop was modeled without considering the cropping system; unsorted grain yield 3.0 t/ha, standard moisture content of 14%; 75% protein crop-25% cereal; intercrop aim: to produce high-protein wheat; intercrop is non-irrigated; intercrop is fertilized; intercrop system built by a group of experts. |
| Winter faba bean, from intercrop, organic, system no. 3, at farm gate/FR U | Comment: These data represent a typical case and are not representative of a national or regional average  system no. 3: Crop from winter wheat and winter faba bean intercrop system no. 2; intercrop was modeled without considering the cropping system; unsorted grain yield 2.5 t/ha, standard moisture content of 14%; 63% protein crop-37% cereal; intercrop aim: to produce protein crop; intercrop is non-irrigated; intercrop is non-fertilized; intercrop system built by a group of experts. |
| Winter faba bean, organic, system no. 1, at farm gate/FR U | Comment: These data represent a typical case and are not representative of a national or regional average  system no. 1: Crop from cropping system no. 6; year 3 in crop rotation; grain yield 2.5 t/ha, standard moisture content of 15%; located in Pays de la Loire, France; production potential of the soil is good; cropping system is irrigated; cropping system is fertilized 2 years out of 3 with compost from poultry manure and dried laying-hen droppings; data are from a typical case from the CASDAR project ProtéAB. |
| Winter faba bean, organic, system no. 2, at farm gate/FR U | Comment: These data represent a typical case and are not representative of a national or regional average  system no. 2: Crop from cropping system no. 3; year 7 in crop rotation; grain yield 2.7 t/ha, standard moisture content of 15%; located in Ile-de-France, France; production potential of the soil is good; cropping system is non-irrigated; cropping system is fertilized 4 years out of 10 with compost from poultry manure and concentrated residues of beetroot distillation; data are from a typical case from the CASDAR project RotAB. |
| Winter faba bean, organic, system no. 3, at farm gate/FR U | Comment: These data represent a typical case and are not representative of a national or regional average  system no. 3: Crop from cropping system no. 8; year 6 in crop rotation; grain yield 2.9 t/ha, standard moisture content of 15%; located in Poitou-Charentes, France; production potential of the soil is average/good; cropping system is irrigated; cropping system is fertilized 3 years out of 9 with compost from poultry manure; data are from a typical case from the CASDAR project ProtéAB. |
| Winter faba bean, organic, system no. 4, at farm gate/FR U | Comment: These data represent a typical case and are not representative of a national or regional average  system no. 4: Crop from cropping system no. 9; year 6 in crop rotation; grain yield 2.1 t/ha, standard moisture content of 15%; located in Poitou-Charentes, France; production potential of the soil is average/good; cropping system is non-irrigated; cropping system is fertilized 2 years out of 9 with compost from poultry manure; data are from a typical case from the CASDAR project ProtéAB. |
| Winter pea, from intercrop, organic, system no. 1, at farm gate/FR U | Comment: These data represent a typical case and are not representative of a national or regional average  system no. 1: Crop from spring pea and spring wheat intercrop system no. 1; intercrop was modeled without considering the cropping system; sorted grain yield 1.0 t/ha, standard moisture content of 14%; intercrop is composed of 22% protein crop-78% cereal; intercrop aim: to produce animal feed; intercrop is non-irrigated; intercrop is fertilized; intercrop system built by a group of experts. |
| Winter pea, from intercrop, organic, system no. 2, at farm gate/FR U | Comment: These data represent a typical case and are not representative of a national or regional average  system no. 2: Crop from triticale grain and winter pea intercrop system no. 2; intercrop from cropping system no. 2; year 9 in crop rotation; sorted grain yield 1.3 t/ha, standard moisture content of 14%; intercrop is composed of 67.5% protein crop-32.5% cereal; located in Brittany, France; production potential of the soil is average/good; intercrop is non-irrigated; intercrop is non-fertilized; cropping system is fertilized 3 years out of 9 with bovine manure and compost from poultry manure; data are from a typical case from the CASDAR project ProtéAB. |
| Winter pea, from intercrop, organic, system no. 3, at farm gate/FR U | Comment: These data represent a typical case and are not representative of a national or regional average  system no. 3: Crop from triticale grain and winter pea intercrop system no. 3; intercrop from cropping system no. 1; year 6 in crop rotation; grain yield 0.9 t/ha, standard moisture content of 14%; intercrop is composed of 36% protein crop-64% cereal; located in Bourgogne, France; production potential of the soil is low/average; intercrop is non-irrigated; intercrop is non-fertilized; cropping system is non-irrigated; cropping system is fertilized 3 years out of 7 with bovine manure; data are from a typical case from the CASDAR project ProtéAB. |
| Winter pea, from intercrop, organic, system no. 4, at farm gate/FR U | Comment: These data represent a typical case and are not representative of a national or regional average  system no. 4: Crop from Triticale grain and winter pea intercrop system no. 4; intercrop from cropping system no. 7; year 2 in crop rotation; grain yield 8.7 t/ha, standard moisture content of 14%; intercrop is composed of 77% protein crop-23% cereal; located in Pays de la Loire, France; production potential of the soil is average; intercrop is non-irrigated; intercrop is non-fertilized; cropping system is non-irrigated; cropping system is fertilized 3 years out of 5 with compost from poultry manure; data are from a typical case from the CASDAR project ProtéAB. |
| Winter pea, from intercrop, organic, system no. 5, at farm gate/FR U | Comment: These data represent a typical case and are not representative of a national or regional average  system no. 5: Crop from winter barley and winter pea intercrop system no. 1; intercrop was modeled without considering the cropping system; unsorted grain yield 2.0 t/ha, standard moisture content of 14%; 50% protein crop-50% cereal; intercrop aim: to produce protein crop; intercrop is non-irrigated; intercrop is non-fertilized; intercrop system built by a group of experts. |
| Winter pea, from intercrop, organic, system no. 6, at farm gate/FR U | Comment: These data represent a typical case and are not representative of a national or regional average  system no. 6: Crop from winter wheat and winter pea intercrop system no. 1; intercrop was modeled without considering the cropping system; unsorted grain yield 3.0 t/ha, standard moisture content of 14%; 75% protein crop-25% cereal; intercrop aim: to produce high-protein wheat; intercrop is non-irrigated; intercrop is fertilized; intercrop system built by a group of experts. |
| Winter pea, from intercrop, organic, system no. 7, at farm gate/FR U | Comment: These data represent a typical case and are not representative of a national or regional average  system no. 7: Crop from winter wheat and winter pea intercrop system no. 2; intercrop was modeled without considering the cropping system; unsorted grain yield 2.0 t/ha, standard moisture content of 14%; 50% protein crop-50% cereal; intercrop aim: to produce protein crop; intercrop is non-irrigated; intercrop is non-fertilized; intercrop system built by a group of experts. |
| Sunflower grain, organic, system no. 1, at farm gate/FR U | Comment: These data represent a typical case and are not representative of a national or regional average  system no. 1: Crop from cropping system no. 5; year 5 in crop rotation; grain yield 1.7 t/ha, standard moisture content of 9%; located in Midi-Pyrénées, France; production potential of the soil is average; cropping system is non-irrigated; cropping system is fertilized 3 years out of 6 with 9-5-0 fertilizer usable in organic agriculture; data are from a typical case from Agribenchmark. |
| Sunflower grain, organic, system no. 2, at farm gate/FR U | Comment: These data represent a typical case and are not representative of a national or regional average  system no. 2: Crop from cropping system no. 7; year 5 in crop rotation; grain yield 2.3 t/ha, standard moisture content of 9%; located in Pays de la Loire, France; production potential of the soil is average; cropping system is non-irrigated; cropping system is fertilized 3 years out of 5 with compost from poultry manure; data are from a typical case from the CASDAR project ProtéAB. |
| Sunflower grain, organic, system no. 3, at farm gate/FR U | Comment: These data represent a typical case and are not representative of a national or regional average  system no. 3: Crop from cropping system no. 8; year 8 in crop rotation; grain yield 2.0 t/ha, standard moisture content of 9%; located in Poitou-Charentes, France; production potential of the soil is average/good; cropping system is irrigated; cropping system is fertilized 3 years out of 9 with compost from poultry manure; data are from a typical case from the CASDAR project ProtéAB. |
| Sunflower grain, organic, system no. 4, at farm gate/FR U | Comment: These data represent a typical case and are not representative of a national or regional average  system no. 4: Crop from cropping system no. 9; year 8 in crop rotation; grain yield 2.0 t/ha, standard moisture content of 9%; located in Poitou-Charentes, France; production potential of the soil is average/good; cropping system is non-irrigated; cropping system is fertilized 2 years out of 9 with compost from poultry manure; data are from a typical case from the CASDAR project ProtéAB. |
| Sunflower grain, organic, system no. 5, at farm gate/FR U | Comment: These data represent a typical case and are not representative of a national or regional average  system no. 5: Crop from cropping system no. 11; year 6 in crop rotation; grain yield 2.0 t/ha, standard moisture content of 9%; located in Rhône-Alpes, France; production potential of the soil is good; cropping system is non-irrigated; cropping system is fertilized 2 years out of 6 with potassium fertilizer usable in organic agriculture and compost from poultry manure; data are from a typical case from the CASDAR project OléAB. |
| Winter rapeseed, organic, at farm gate/FR U | Comment : This life cycle inventory was created based on literature and expert knowledge. Yield: 2.2 t/ha, standard moisture content of 9%.  Sampling procedure: Technical data come from several sources: ITAB, Opaba, Agridea (2009), Chambre d'agriculture Rhône Alpes (2012) and Agrobio Bretagne (2009) |
| Alfalfa, organic, system no. 1, at farm gate/FR U | Comment: These data represent a typical case and are not representative of a national or regional average  system no. 1: Forage from cropping system no. 1; years 1-3 in crop rotation; yield 5 t of dry matter/ha.year; located in Bourgogne, France; production potential of the soil is low/average; cropping system is non-irrigated; cropping system is fertilized 3 years out of 7 with bovine manure; data are from a typical case from the CASDAR project ProtéAB. |
| Alfalfa, organic, system no. 2, at farm gate/FR U | Comment: These data represent a typical case and are not representative of a national or regional average  system no. 2: Forage from cropping system no. 3; years 1-3 in crop rotation; yield 9.4 t of dry matter/ha.year; located in Ile-de-France, France; production potential of the soil is good; cropping system is non-irrigated; cropping system is fertilized 4 years out of 10 with compost from poultry manure and concentrated residues of beetroot distillation; data are from a typical case from the CASDAR project RotAB. |
| Alfalfa, organic, system no. 3, at farm gate/FR U | Comment: These data represent a typical case and are not representative of a national or regional average  system no. 3: Forage from cropping system no. 8; years 1-3 in crop rotation; yield 6.67 t of dry matter/ha.year; located in Poitou-Charentes, France; production potential of the soil is average/good; cropping system is irrigated; cropping system is fertilized 3 years out of 9 with compost from poultry manure; data are from a typical case from the CASDAR project ProtéAB. |
| Alfalfa, organic, system no. 4, at farm gate/FR U | Comment: These data represent a typical case and are not representative of a national or regional average  system no. 4: Forage from cropping system no. 9; years 1-3 in crop rotation; yield 7.67 t of dry matter/ha.year; located in Poitou-Charentes, France; production potential of the soil is average/good; cropping system is non-irrigated; cropping system is fertilized 2 years out of 9 with compost from poultry manure; data are from a typical case from the CASDAR project ProtéAB. |
| Alfalfa, organic, system no. 5, at farm gate/FR U | Comment: These data represent a typical case and are not representative of a national or regional average  system no. 5: Forage from cropping system no. 11; years 1-3 in crop rotation; yield 3.5 t of dry matter/ha.year; located in Rhône-Alpes, France; production potential of the soil is good; cropping system is non-irrigated; cropping system is fertilized 2 years out of 6 with potassium fertilizer usable in organic agriculture and compost from poultry manure; data are from a typical case from the CASDAR project OléAB. |
| Baled hay, temporary meadow, organic, system no. 5, at farm gate/FR U | Comment: These data represent a typical case and are not representative of a national or regional average  system no. 5: Forage from cropping system no. 2; years 1-3 in crop rotation; forage yield 3.67 t of dry matter/ha.year; located in Brittany, France; production potential of the soil is average/good; non-irrigated; fertilized 3 years out of 9 with bovine manure and compost from poultry manure; data are from a typical case from the CASDAR project ProtéAB. |
| Silage maize, organic, system no. 1, at farm gate/FR U | Comment: This life cycle inventory was created based on literature and expert knowledge. Yield: 11.6 t/ha, standard moisture content of 28%.  Sampling procedure: Personal communication of H. Chambaut (IDELE) for yield, fertilisation and crop rotation.  Technical data from ITAB-Chambre d'agriculture Pays de la Loire et Manche-Agribio 04. |
| Sorghum, organic, system no. 1, at farm gate/FR U | Comment: These data represent a typical case and are not representative of a national or regional average  system no. 1: Forage from cropping system no. 1; year 7 in crop rotation; forage yield 1.4 t of dry matter/ha.year; located in Bourgogne, France; production potential of the soil is low/average; non-irrigated; fertilized 3 years out of 7 with bovine manure; data are from a typical case from the CASDAR project ProtéAB. |
| Broiler, organic, system no. 1, at farm gate/FR U | Comment: These data are based on a typical case and are not representative of a national or regional average.  system no. 1: broiler age: 86,4 days; broiler weight: 2,28 kg; broiler feed composed at 95% of organic products; located in west of France. |
| Broiler, organic, system no. 2, at farm gate/FR U | Comment: These data are based on a typical case and are not representative of a national or regional average.  system no. 2: broiler age: 86,4 days; broiler weight: 2,28 kg; broiler feed composed at 100% of organic products; located in west of France. |
| Cull hen, organic, system no. 1, at farm gate/FR U | Comment: These data are based on a typical case and are not representative of a national or regional average.  system no. 1: number of eggs per hen: 280 for one year; egg weight: 64.5 g; hen feed composed at 95% of organic products; located in west of France. |
| Cull hen, organic, system no. 2, at farm gate/FR U | Comment: These data are based on a typical case and are not representative of a national or regional average.  system no. 2: number of eggs per hen: 280 for one year; egg weight: 64.5 g; hen feed composed at 100% of organic products; located in west of France. |
| Egg, organic, system no. 1, at farm gate/FR U | Comment: These data are based on a typical case and are not representative of a national or regional average.  system no. 1: number of eggs per hen: 280 for one year; egg weight: 64.5 g; hen feed composed at 95% of organic products; located in west of France. |
| Egg, organic, system no. 2, at farm gate/FR U | Comment: These data are based on a typical case and are not representative of a national or regional average.  system no. 2: number of eggs per hen: 280 for one year; egg weight: 64.5 g; hen feed composed at 100% of organic products; located in west of France. |
| Calf, 14 days old, organic, milk system no. 1, at farm gate/FR U | Comment: These data are based on a typical case and are not representative of a national or regional average.  system no. 1: 3300 L of milk produced per ha of forage; 5880 L per cow and per year; low mountain region, east of France; 0% of maize per hectare of forage area; 200 g/L of concentrated feed; autonomy in contrasted feed: 52%; Typical case study "Auvergne-Lozère" based on data collected by INOSYS. |
| Calf, 14 days old, organic, milk system no. 2, at farm gate/FR U | Comment: These data are based on a typical case and are not representative of a national or regional average.  system no. 2: 3400 L of milk produced per ha of forage; 5600 L per cow and per year; lowland region, continental plain, east of France; 0% of maize per hectare of forage area; 178 g/L of concentrated feed; autonomy in contrasted feed: 93%; Typical case study "Est" based on data collected by INOSYS. |
| Calf, 14 days old, organic, milk system no. 3, at farm gate/FR U | Comment: These data are based on a typical case and are not representative of a national or regional average.  system no. 3: 4350 L of milk produced per ha of forage; 4610 L per cow and per year; lowland region, oceanic plain, weast of France; 0% of maize per hectare of forage area; 100 g/L of concentrated feed; autonomy in contrasted feed: 27%; Individual case study "Orne" based on data collected on the Optialibio farm. |
| Calf, 14 days old, organic, milk system no. 4, at farm gate/FR U | Comment: These data are based on a typical case and are not representative of a national or regional average.  system no. 4: 4850 L of milk produced per ha of forage; 6510 L per cow and per year; lowland region, oceanic plain, weast of France; 17% of maize per hectare of forage area; 142 g/L of concentrated feed; autonomy in contrasted feed: 80%; Typical case study "Pays de la Loire" based on data collected by INOSYS. |
| Calf, 14 days old, organic, milk system no. 5, at farm gate/FR U | Comment: These data are based on a typical case and are not representative of a national or regional average.  system no. 5: 6000 L of milk produced per ha of forage; 6750 L per cow and per year; lowland region, oceanic plain, weast of France; 0% of maize per hectare of forage area; 102 g/L of concentrated feed; autonomy in contrasted feed: 100%; Typical case study "Bretagne" based on data collected by INOSYS. |
| Cull cow, organic, milk system no. 1, at farm gate/FR U | Comment: These data are based on a typical case and are not representative of a national or regional average.  system no. 1: 3300 L of milk produced per ha of forage; 5880 L per cow and per year; low mountain region, east of France; 0% of maize per hectare of forage area; 200 g/L of concentrated feed; autonomy in contrasted feed: 52%; Typical case study "Auvergne-Lozère" based on data collected by INOSYS. |
| Cull cow, organic, milk system no. 2, at farm gate/FR U | Comment: These data are based on a typical case and are not representative of a national or regional average.  system no. 2: 3400 L of milk produced per ha of forage; 5600 L per cow and per year; lowland region, continental plain, east of France; 0% of maize per hectare of forage area; 178 g/L of concentrated feed; autonomy in contrasted feed: 93%; Typical case study "Est" based on data collected by INOSYS. |
| Cull cow, organic, milk system no. 3, at farm gate/FR U | Comment: These data are based on a typical case and are not representative of a national or regional average.  system no. 3: 4350 L of milk produced per ha of forage; 4610 L per cow and per year; lowland region, oceanic plain, weast of France; 0% of maize per hectare of forage area; 100 g/L of concentrated feed; autonomy in contrasted feed: 27%; Individual case study "Orne" based on data collected on the Optialibio farm. |
| Cull cow, organic, milk system no. 4, at farm gate/FR U | Comment: These data are based on a typical case and are not representative of a national or regional average.  system no. 4: 4850 L of milk produced per ha of forage; 6510 L per cow and per year; lowland region, oceanic plain, weast of France; 17% of maize per hectare of forage area; 142 g/L of concentrated feed; autonomy in contrasted feed: 80%; Typical case study "Pays de la Loire" based on data collected by INOSYS. |
| Cull cow, organic, milk system no. 5, at farm gate/FR U | Comment: These data are based on a typical case and are not representative of a national or regional average.  system no. 5: 6000 L of milk produced per ha of forage; 6750 L per cow and per year; lowland region, oceanic plain, weast of France; 0% of maize per hectare of forage area; 102 g/L of concentrated feed; autonomy in contrasted feed: 100%; Typical case study "Bretagne" based on data collected by INOSYS. |
| Cow milk, organic, system no. 1, at farm gate/FR U | Comment: These data are based on a typical case and are not representative of a national or regional average.  system no. 1: 3300 L of milk produced per ha of forage; 5880 L per cow and per year; low mountain region, east of France; 0% of maize per hectare of forage area; 200 g/L of concentrated feed; autonomy in contrasted feed: 52%; Typical case study "Auvergne-Lozère" based on data collected by INOSYS. |
| Cow milk, organic, system no. 2, at farm gate/FR U | Comment: These data are based on a typical case and are not representative of a national or regional average.  system no. 2: 3400 L of milk produced per ha of forage; 5600 L per cow and per year; lowland region, continental plain, east of France; 0% of maize per hectare of forage area; 178 g/L of concentrated feed; autonomy in contrasted feed: 93%; Typical case study "Est" based on data collected by INOSYS. |
| Cow milk, organic, system no. 3, at farm gate/FR U | Comment: These data are based on a typical case and are not representative of a national or regional average.  system no. 3: 4350 L of milk produced per ha of forage; 4610 L per cow and per year; lowland region, oceanic plain, weast of France; 0% of maize per hectare of forage area; 100 g/L of concentrated feed; autonomy in contrasted feed: 27%; Individual case study "Orne" based on data collected on the Optialibio farm. |
| Cow milk, organic, system no. 4, at farm gate/FR U | Comment: These data are based on a typical case and are not representative of a national or regional average.  system no. 4: 4850 L of milk produced per ha of forage; 6510 L per cow and per year; lowland region, oceanic plain, weast of France; 17% of maize per hectare of forage area; 142 g/L of concentrated feed; autonomy in contrasted feed: 80%; Typical case study "Pays de la Loire" based on data collected by INOSYS. |
| Cow milk, organic, system no. 5, at farm gate/FR U | Comment: These data are based on a typical case and are not representative of a national or regional average.  system no. 5: 6000 L of milk produced per ha of forage; 6750 L per cow and per year; lowland region, oceanic plain, weast of France; 0% of maize per hectare of forage area; 102 g/L of concentrated feed; autonomy in contrasted feed: 100%; Typical case study "Bretagne" based on data collected by INOSYS. |
| Cull ewe, organic, system no. 1, at farm gate/FR U | Comment: These data are based on a typical case and are not representative of a national or regional average.  system no. 1: 31,5 kg of lamb produced per ewe; weight of lamb before slaughter: 41.3kg; weight of ewe before slaughter: 70kg; wool produced per ewe: 2.6 kg; race: Berrichon du Cher; located in Centre, France; finishing of lamb in pasture; data come from a typical case from the Agneau bio project. |
| Cull ewe, organic, system no. 2, at farm gate/FR U | Comment: These data are based on a typical case and are not representative of a national or regional average.  system no. 2: 43 kg of lamb produced per ewe; weight of lamb before slaughter: 37.5kg; weight of ewe before slaughter: 70kg; wool produced per ewe: 3.5 kg; race: Texel; located in Lorraine, France; finishing of lamb in pasture; data come from a typical case from the Agneau bio project. |
| Cull ewe, organic, system no. 3, at farm gate/FR U | Comment: These data are based on a typical case and are not representative of a national or regional average.  system no. 3: 28 kg of lamb produced per ewe; weight of lamb before slaughter: 33.3kg; weight of ewe before slaughter: 60kg; wool produced per ewe: 1.96 kg; race: Mérinos d’Arles; located in Provence-Alpes-Côte d'Azur, France; pastoral system; data come from a typical case from the Agneau bio project. |
| Lamb, organic, system no. 1, at farm gate/FR U | Comment: These data are based on a typical case and are not representative of a national or regional average.  system no. 1: 31,5 kg of lamb produced per ewe; weight of lamb before slaughter: 41.3kg; weight of ewe before slaughter: 70kg; wool produced per ewe: 2.6 kg; race: Berrichon du Cher; located in Centre, France; finishing of lamb in pasture; data come from a typical case from the Agneau bio project. |
| Lamb, organic, system no. 2, at farm gate/FR U | Comment: These data are based on a typical case and are not representative of a national or regional average.  system no. 2: 43 kg of lamb produced per ewe; weight of lamb before slaughter: 37.5kg; weight of ewe before slaughter: 70kg; wool produced per ewe: 3.5 kg; race: Texel; located in Lorraine, France; finishing of lamb in pasture; data come from a typical case from the Agneau bio project. |
| Lamb, organic, system no. 3, at farm gate/FR U | Comment: These data are based on a typical case and are not representative of a national or regional average.  system no. 3: 28 kg of lamb produced per ewe; weight of lamb before slaughter: 33.3kg; weight of ewe before slaughter: 60kg; wool produced per ewe: 1.96 kg; race: Mérinos d’Arles; located in Provence-Alpes-Côte d'Azur, France; pastoral system; data come from a typical case from the Agneau bio project. |
| Wool, organic, system no. 1, at farm gate/FR U | Comment: These data are based on a typical case and are not representative of a national or regional average.  system no. 1: 31,5 kg of lamb produced per ewe; weight of lamb before slaughter: 41.3kg; weight of ewe before slaughter: 70kg; wool produced per ewe: 2.6 kg; race: Berrichon du Cher; located in Centre, France; finishing of lamb in pasture; data come from a typical case from the Agneau bio project. |
| Wool, organic, system no. 2, at farm gate/FR U | Comment: These data are based on a typical case and are not representative of a national or regional average.  system no. 2: 43 kg of lamb produced per ewe; weight of lamb before slaughter: 37.5kg; weight of ewe before slaughter: 70kg; wool produced per ewe: 3.5 kg; race: Texel; located in Lorraine, France; finishing of lamb in pasture; data come from a typical case from the Agneau bio project. |
| Wool, organic, system no. 3, at farm gate/FR U | Comment: These data are based on a typical case and are not representative of a national or regional average.  system no. 3: 28 kg of lamb produced per ewe; weight of lamb before slaughter: 33.3kg; weight of ewe before slaughter: 60kg; wool produced per ewe: 1.96 kg; race: Mérinos d’Arles; located in Provence-Alpes-Côte d'Azur, France; pastoral system; data come from a typical case from the Agneau bio project. |
| Cull sow, organic, national average, at farm gate/FR U | Comment: these data represent a national average. Breeder/fattener system; 1930 kg of fattened pig produced per sow and per year; sows are raised outdoors and weaned piglets and pigs are raised indoors; buildings with straw; feeds are 40% produced on farm and 60% purchased; located in Nouvelle-Aquitaine, France. |
| Cull sow, organic, system no. 1, at farm gate/FR U | Comment: These data are based on a typical case and are not representative of a national or regional average.  system no. 1: breeder/fattener system; 1980 kg of fattened pig produced per sow and per year; sows are raised outdoors and weaned piglets and pigs are raised indoors; buildings with straw; feeds are produced on farm; located in Nouvelle-Aquitaine, France. |
| Cull sow, organic, system no. 2, at farm gate/FR U | Comment: These data are based on a typical case and are not representative of a national or regional average.  system no. 2: breeder/fattener system; 1729 kg of fattened pig produced per sow and per year; sows are raised outdoors and weaned piglets and pigs are raised indoors; buildings with straw; feeds are produced on farm; located in Nouvelle-Aquitaine, France. |
| Cull sow, organic, system no. 3, at farm gate/FR U | Comment: These data are based on a typical case and are not representative of a national or regional average.  system no. 3: breeder/fattener system; 2286 kg of fattened pig produced per sow and per year; sows are raised outdoors and weaned piglets and pigs are raised indoors; buildings with straw; feeds are purchased; located in Nouvelle-Aquitaine, France. |
| Cull sow, organic, system no. 4, at farm gate/FR U | Comment: These data are based on a typical case and are not representative of a national or regional average.  system no. 4: breeder/fattener system; 1641 kg of fattened pig produced per sow and per year; sows, weaned piglets and pigs are raised indoors; buildings with straw; feeds are purchased; located in Nouvelle-Aquitaine, France. |
| Pig, organic, national average, at farm gate/FR U | Comment: these data represent a national average. breeder/fattener system; 1930 kg of fattened pig produced per sow and per year; sows are raised outdoors and weaned piglets and pigs are raised indoors; buildings with straw; feeds are 40% produced on farm and 60% purchased; located in Nouvelle-Aquitaine, France. |
| Pig, organic, system no. 1, at farm gate/FR U | Comment: These data are based on a typical case and are not representative of a national or regional average.  system no. 1: breeder/fattener system; 1980 kg of fattened pig produced per sow and per year; sows are raised outdoors and weaned piglets and pigs are raised indoors; buildings with straw; feeds are produced on farm; located in Nouvelle-Aquitaine, France. |
| Pig, organic, system no. 2, at farm gate/FR U | Comment: These data are based on a typical case and are not representative of a national or regional average.  system no. 2: breeder/fattener system; 1729 kg of fattened pig produced per sow and per year; sows are raised outdoors and weaned piglets and pigs are raised indoors; buildings with straw; feeds are produced on farm; located in Nouvelle-Aquitaine, France. |
| Pig, organic, system no. 3, at farm gate/FR U | Comment: These data are based on a typical case and are not representative of a national or regional average.  system no. 3: breeder/fattener system; 2286 kg of fattened pig produced per sow and per year; sows are raised outdoors and weaned piglets and pigs are raised indoors; buildings with straw; feeds are purchased; located in Nouvelle-Aquitaine, France. |
| Pig, organic, system no. 4, at farm gate/FR U | Comment: These data are based on a typical case and are not representative of a national or regional average.  system no. 4: breeder/fattener system; 1641 kg of fattened pig produced per sow and per year; sows, weaned piglets and pigs are raised indoors; buildings with straw; feeds are purchased; located in Nouvelle-Aquitaine, France. |
| Cull cow, organic, suckler cow system, system no. 1, at farm gate/FR U | Comment: These data are based on a typical case and are not representative of a national or regional average.  Subsystem “breeding” of system no. 1: breeding-fattening herd with calves raised under the mother, described as the Charolais breed in a western oceanic climate. At the farm scale, the forage area contains 92% grass, mainly temporary grassland. Cattle are managed in two batches, with two-thirds of the cows calving in the fall. The age at first calving is 35 months. Calves are sold at 125 days of age for slaughter (daily gain of 1080 g/d). Overall productivity is 245 kg live weight per livestock unit. Typical case study "Brittany" based on data collected by the INOSYS Réseaux d’élevage, a collaboration between French Chambers of Agriculture and the French Livestock Institute. |
| Cull cow, organic, suckler cow system, system no. 2, at farm gate/FR U | Comment: These data are based on a typical case and are not representative of a national or regional average.  Subsystem “breeding” of system no. 2: breeding herd, described as the Charolais breed in a western oceanic climate. At the farm scale, the agricultural area contains 90% grass, mainly permanent grassland. Cattle are managed in two batches, with 75% of the cows calving in February and 25% in September. Age at first calving is 36 months. Runner calves are sold at 270-280 kg of live weight/head. Overall productivity is 265 kg live weight per livestock unit. Typical case study "Pays de la Loire-Deux Sevres" based on data collected by the INOSYS Réseaux d’élevage, a collaboration between French Chambers of Agriculture and the French Livestock Institute. |
| Cull cow, organic, suckler cow system, system no. 3, at farm gate/FR U | Comment: These data are based on a typical case and are not representative of a national or regional average.  Subsystem “breeding” of system no. 3: breeding-fattening herd that produces male cattles, described as the Blonde d’Aquitaine breed in a mixed farming zone in northern France. At the farm level, the forage area contains 100% grass, and the agricultural area contains 54% grain crops. There is only one calving period (March-May). Age at first calving is 35 months. Cattle are sold at 450 kg carcass weight/head. Overall productivity is 332 kg live weight per livestock unit. Typical case study "Picardie" based on data collected by the INOSYS Réseaux d’élevage, a collaboration between French Chambers of Agriculture and the French Livestock Institute. |
| Cull cow, organic, suckler cow system, system no. 4, at farm gate/FR U | Comment: These data are based on a typical case and are not representative of a national or regional average.  Subsystem “breeding” of system no. 4: breeding-fattening herd that produces male and female cattle, described as the Charolaise breed and produced for direct sale in the Rhône Alpes region. At the farm level, the forage area contains 100% grass, mostly permanent grassland. There is only one calving period (January-April). Age at first calving is 36 months. Male and female cattle are sold at 440 and 350-380 kg carcass weight/head, respectively. Overall productivity is 225 kg live weight per livestock unit. Typical case study "Direct sales" based on data collected by the INOSYS Réseaux d’élevage, a collaboration between French Chambers of Agriculture and the French Livestock Institute. |
| Fattening cattle, female, organic, beef fattening system no. 1, at farm gate/FR U | Comment: These data are based on a typical case and are not representative of a national or regional average.  Subsystem “fattening” of system no. 1: breeding-fattening herd with calves raised under the mother, described as the Charolais breed in a western oceanic climate. At the farm scale, the forage area contains 92% grass, mainly temporary grassland. Cattle are managed in two batches, with two-thirds of the cows calving in the fall. The age at first calving is 35 months. Calves are sold at 125 days of age for slaughter (daily gain of 1080 g/d). Overall productivity is 245 kg live weight per livestock unit. Typical case study "Brittany" based on data collected by the INOSYS Réseaux d’élevage, a collaboration between French Chambers of Agriculture and the French Livestock Institute. |
| Fattening cattle, female, organic, beef fattening system no. 2, at farm gate/FR U | Comment: These data are based on a typical case and are not representative of a national or regional average.  Subsystem “fattening” of system no. 2: breeding herd, described as the Charolais breed in a western oceanic climate. At the farm scale, the agricultural area contains 90% grass, mainly permanent grassland. Cattle are managed in two batches, with 75% of the cows calving in February and 25% in September. Age at first calving is 36 months. Runner calves are sold at 270-280 kg of live weight/head. Overall productivity is 265 kg live weight per livestock unit. Typical case study "Pays de la Loire-Deux Sevres" based on data collected by the INOSYS Réseaux d’élevage, a collaboration between French Chambers of Agriculture and the French Livestock Institute. |
| Fattening cattle, female, organic, beef fattening system no. 3, at farm gate/FR U | Comment: These data are based on a typical case and are not representative of a national or regional average.  Subsystem “fattening” of system no. 3: breeding-fattening herd that produces male cattles, described as the Blonde d’Aquitaine breed in a mixed farming zone in northern France. At the farm level, the forage area contains 100% grass, and the agricultural area contains 54% grain crops. There is only one calving period (March-May). Age at first calving is 35 months. Cattle are sold at 450 kg carcass weight/head. Overall productivity is 332 kg live weight per livestock unit. Typical case study "Picardie" based on data collected by the INOSYS Réseaux d’élevage, a collaboration between French Chambers of Agriculture and the French Livestock Institute. |
| Fattening cattle, female, organic, beef fattening system no. 4, at farm gate/FR U | Comment: These data are based on a typical case and are not representative of a national or regional average.  Subsystem “fattening” of system no. 4: breeding-fattening herd that produces male and female cattle, described as the Charolaise breed and produced for direct sale in the Rhône Alpes region. At the farm level, the forage area contains 100% grass, mostly permanent grassland. There is only one calving period (January-April). Age at first calving is 36 months. Male and female cattle are sold at 440 and 350-380 kg carcass weight/head, respectively. Overall productivity is 225 kg live weight per livestock unit. Typical case study "Direct sales" based on data collected by the INOSYS Réseaux d’élevage, a collaboration between French Chambers of Agriculture and the French Livestock Institute. |
| Fattening cattle, male, organic, beef fattening system no. 3, at farm gate/FR U | Comment: These data are based on a typical case and are not representative of a national or regional average.  Subsystem “fattening” of system no. 3: breeding-fattening herd that produces male cattles, described as the Blonde d’Aquitaine breed in a mixed farming zone in northern France. At the farm level, the forage area contains 100% grass, and the agricultural area contains 54% grain crops. There is only one calving period (March-May). Age at first calving is 35 months. Cattle are sold at 450 kg carcass weight/head. Overall productivity is 332 kg live weight per livestock unit. Typical case study "Picardie" based on data collected by the INOSYS Réseaux d’élevage, a collaboration between French Chambers of Agriculture and the French Livestock Institute. |
| Fattening cattle, male, organic, beef fattening system no. 4, at farm gate/FR U | Comment: These data are based on a typical case and are not representative of a national or regional average.  Subsystem “fattening” of system no. 4: breeding-fattening herd that produces male and female cattle, described as the Charolaise breed and produced for direct sale in the Rhône Alpes region. At the farm level, the forage area contains 100% grass, mostly permanent grassland. There is only one calving period (January-April). Age at first calving is 36 months. Male and female cattle are sold at 440 and 350-380 kg carcass weight/head, respectively. Overall productivity is 225 kg live weight per livestock unit. Typical case study "Direct sales" based on data collected by the INOSYS Réseaux d’élevage, a collaboration between French Chambers of Agriculture and the French Livestock Institute. |
| Calf, weaned, 4 months old, organic, suckler cow system no. 1, at farm gate/FR U | Comment: These data are based on a typical case and are not representative of a national or regional average.  Subsystem “breeding” of system no. 1: breeding-fattening herd with calves raised under the mother, described as the Charolais breed in a western oceanic climate. At the farm scale, the forage area contains 92% grass, mainly temporary grassland. Cattle are managed in two batches, with two-thirds of the cows calving in the fall. The age at first calving is 35 months. Calves are sold at 125 days of age for slaughter (daily gain of 1080 g/d). Overall productivity is 245 kg live weight per livestock unit. Typical case study "Brittany" based on data collected by the INOSYS Réseaux d’élevage, a collaboration between French Chambers of Agriculture and the French Livestock Institute. |
| Calf, weaned, 8 months old, organic, suckler cow system no. 2, at farm gate/FR U | Comment: These data are based on a typical case and are not representative of a national or regional average.  Subsystem “breeding” of system no. 2: breeding herd, described as the Charolais breed in a western oceanic climate. At the farm scale, the agricultural area contains 90% grass, mainly permanent grassland. Cattle are managed in two batches, with 75% of the cows calving in February and 25% in September. Age at first calving is 36 months. Runner calves are sold at 270-280 kg of live weight/head. Overall productivity is 265 kg live weight per livestock unit. Typical case study "Pays de la Loire-Deux Sevres" based on data collected by the INOSYS Réseaux d’élevage, a collaboration between French Chambers of Agriculture and the French Livestock Institute. |
| Calf, weaned, 7 months old, organic, suckler cow system no. 3 at farm gate/FR U | Comment: These data are based on a typical case and are not representative of a national or regional average.  Subsystem “breeding” of system no. 3: breeding-fattening herd that produces male cattles, described as the Blonde d’Aquitaine breed in a mixed farming zone in northern France. At the farm level, the forage area contains 100% grass, and the agricultural area contains 54% grain crops. There is only one calving period (March-May). Age at first calving is 35 months. Cattle are sold at 450 kg carcass weight/head. Overall productivity is 332 kg live weight per livestock unit. Typical case study "Picardie" based on data collected by the INOSYS Réseaux d’élevage, a collaboration between French Chambers of Agriculture and the French Livestock Institute. |
| Calf, weaned, 9 months old, organic, suckler cow system no. 4, at farm gate/FR U | Comment: These data are based on a typical case and are not representative of a national or regional average.  Subsystem “breeding” of system no. 4: breeding-fattening herd that produces male and female cattle, described as the Charolaise breed and produced for direct sale in the Rhône Alpes region. At the farm level, the forage area contains 100% grass, mostly permanent grassland. There is only one calving period (January-April). Age at first calving is 36 months. Male and female cattle are sold at 440 and 350-380 kg carcass weight/head, respectively. Overall productivity is 225 kg live weight per livestock unit. Typical case study "Direct sales" based on data collected by the INOSYS Réseaux d’élevage, a collaboration between French Chambers of Agriculture and the French Livestock Institute. |

Table S3. Nitrate emissions (kg N-NO_3_/ha) from temporary and permanent grassland for dairy and suckler cows before and after modification

| Grassland | Type of system | Nitrate emissions estimated by the DEAC model | Nitrate emissions after modification |
| --- | --- | --- | --- |
| Permanent grassland system no. 1 | Dairy cows | 13.00 | 2.53 |
| Permanent grassland system no. 2 | Dairy cows | 10.50 | 2.58 |
| Permanent grassland system no. 3 | Dairy cows | 20.00 | 6.21 |
| Temporary grassland system no. 4 | Dairy cows | 29.35 | 1.62 |
| Temporary grassland system no. 5 | Dairy cows | 37.00 | 20.37 |
| Permanent grassland system no. 1 | Beef cows | 9.10 | 4.00 |
| Permanent grassland system no. 2 | Beef cows | 13.52 | 3.09 |
| Permanent grassland system no. 3 | Beef cows | 10.40 | 2.76 |
| Permanent grassland system no. 4 | Beef cows | 10.92 | 2.55 |

Table S4. Characterization method and impact categories. level of recommendation of the impact assessment models: I: recommended and satisfactory, II (recommended but in need of some improvements) [1].

| Characterization method | Impact category | Impact assessment model robustness | Unit |
| --- | --- | --- | --- |
| Ecological Footprint method v2.0 | Climate change | I | kg CO_2_ eq |
|  | Ozone depletion | I | kg CFC11 eq |
|  | Ionizing radiation (human health) | II | kBq U-235 eq |
|  | Photochemical ozone formation (human health) | II | kg NMVOC eq |
|  | Respiratory inorganics | I | disease eq |
|  | Acidification terrestrial and freshwater | II | mol H+ eq |
|  | Eutrophication freshwater | II | kg P eq |
|  | Eutrophication marine | II | kg N eq |
|  | Eutrophication terrestrial | II | mol N eq |

**References**

[1] S. Fazio, V. Castellani, S. Sala, E. Schau, M. Secchi, L. Zampori, Supporting information to the characterisation factors of recommended EF Life Cycle Impact Assessment methods: New models and differences with ILCD, in: Publications Office of the European Union (Ed.), 2018.
